# The making of the ‘useless and pathological’ uterus in Taiwan, 1960s to 1990s

**DOI:** 10.1017/mdh.2020.50

**Published:** 2021-01

**Authors:** Hsiu-Yun Wang

**Affiliations:** School of Medicine, College of Medicine, National Cheng Kung University, 1 University Road, East District, Tainan City, Taiwan

**Keywords:** Useless and pathological uterus, Hysterectomy, Family planning, Cancer prevention, Gender and health, Taiwan

## Abstract

During the 1960s and 1970s, the notion that the uterus is a useless and pathological organ after a woman has had ‘enough’ children emerged alongside news reports of excessive hysterectomy in Taiwan. This notion and hysterectomy became two sides of the same coin, the former pointing to the burden of birth control and cancer risk, and the latter to sterilization and removing cancer risk. I explore how, in post-war Taiwan, the notion became commonplace through the intersection of three historical formations: the medical tradition of employing surgery to manage risk (such as appendectomy for appendicitis), American-dominated family planning projects that intensified the surgical approach and promoted reproductive rationality, and cancer prevention campaigns that helped cultivate a sense of cancer risk. The gender politics operating in the family planning and cancer prevention projects were apparent. The burden of birth control fell mainly on women, and the cancer prevention campaign, centring almost exclusively on early detection of cervical cancer, made cancer into a woman’s disease. I argue that the discourses of reproductive rationality and disease risk were parallel and, in several key ways, intersecting logics that rendered the uterus useless and pathological and then informed surgeons’ practice of hysterectomy. Exploring the ways in which the uterus was envisioned and targeted in the history of medicine in Taiwan, this paper shows overlapping bio-politics in three strands of research in an East Asian context – namely women’s health, family planning and cancer prevention – and offers a case for global comparison.

## H1

In 1971, the Taiwanese obstetrician/gynaecologist, Xian-Jie Meng^1^, wrote in a women’s magazine:

‘Indeed, a woman’s uterus has only one utility, which is giving birth to children. Other than being a place for the fetus to grow, it causes hundreds of harms without one benefit. Especially for women over 35 years old, the uterus is like a time bomb—it can explode with life-threatening diseases at any time’.^2^

Meng was not alone in defining the uterus as a volatile organ that can both nurture and pose a grave threat to life. Dr Xin-Xing Zhang, another of the several ob/gyn doctors who opined on this topic for a popular audience, went so far as to draw an analogy between the uterus and the appendix, arguing that both were useless and pathological.

The notion of the useless and pathological uterus (wuyong qie hui shengbing de zigong; hereafter, wuyong) began to inform doctors’ practice in the 1960s, and it continued well into the 1990s, as indicated in a 1997 study on non-cancer hysterectomy patients in Taipei, which found that 75% of the women were told of the notion of wuyong by their physicians.^3^ Addressed specifically to women who had had ‘enough’ children and were purportedly at risk of cancer, the notion of the useless and pathological uterus, a particular kind of embodied risk, has had profound implications for medical practice, as well as women’s bodies. In the popular literature and women’s personal accounts, the phrase yilao yongyi (one effort, once and for all) rationalised certain actions, most typically surgery. When the veteran uterus was framed as a source of unwanted pregnancy and a health threat, hysterectomy became a convenient – seemingly objective, medico-technological – answer to that threat.

Removal of ‘useless uteruses’ was not a Taiwanese invention; it was a global phenomenon with local variations. The American gynaecologist, Ralph C. Wright, advocated routine hysterectomy in the 1960s, as it ‘demonstrated the value of prophylactic removal of a normal, but useless and potentially cancer-bearing organ’.^4^ J.B. Skelton in 1973 urged the American College of Obstetricians and Gynaecologists to recommend prophylactic elective total hysterectomy and bilateral salpingo-oophorectomy after completion of childbearing as proper preventive medicine.^5^ In the United States, rising significantly during the 1960s and 1970s, the hysterectomy rate reached its peak in 1975, at 725 000; it was the most frequently performed surgery by the 1980s.^6^ ^7^ In 1970s Japan, ob/gyns did something very similar; for instance, more than one thousand women underwent hysterectomy in Fujimi Hospital; when they went to the hospital for regular cancer check-ups, abdominal pain or light bleeding, they were warned that the surgery was urgently needed to avoid cancer.^8^ In early twenty-first century Mexico, hysterectomy was often used as treatment for cervical abnormalities, such as low-grade abnormal cell growth on the cervix.^9^ Recently, Korea’s hysterectomy rate has been at the top among OECD nations, signalling an alarm for the nation.^10^ As a former colony of Japan, and dominated by the US after the Second World War, Taiwan appropriated knowledge and practices from both empires, including prophylactic hysterectomy. Following medical trends in the United States and Japan, surgery stood as the way to free women’s bodies from unwanted pregnancy and cancer risk in the name of medical progress.

Drawing on popular and professional medical writings, women’s published illness stories, and oral interviews with fifteen women who had had a hysterectomy, one midwife and three medical practitioners, this article examines the history of the making of the useless and pathological uterus in Taiwan during the second half of the twentieth century. I explore how, through the cultivation of reproductive rationality and implementation of cancer prevention measures at the interface of government health administration, public health projects and doctor–patient clinical contexts, the uterus became an organ that carried the risks of unwanted pregnancy and cancer. While the medical view of the female body as pathological has a long and well-researched history in the West,^11^ how the wuyong uterus emerged in Taiwan has received little scholarly attention.^12^ I argue that the useless and pathological uterus discourses in Taiwan flourished at the nexus of three interconnected historical formations. First, an emphasis on a surgical approach within the medical profession,^13^ including among ob/gyn practitioners, which had developed in the colonial period, was reinforced after the war by American influence on medical education as it expanded, surgical techniques matured and more surgeons were available. The second was the implementation of a national family planning project, also supported by American funding and American trained professionals, which officially began in 1964 and continued into the late 1980s. It promoted IUDs and sterilization as the best choices for birth control while the uterus increasingly became the main target of control.^14^ The third was large-scale cervical cancer prevention measures in the early 1970s, which included the aggressive transfer of knowledge from the United States (e.g., public health education and Pap smear screening), and the uterus again was the organ of concern. Through these programs and the dissemination of popular health manuals, women were exposed in multiple ways to a rational, scientific approach to birth control and disease prevention and sensitised to a whole range of health risks surrounding unwanted pregnancy and gynaecological diseases.

In short, by focusing on the ways in which the uterus was envisioned and targeted, this paper brings together three strands of research: surgery and women’s health, family planning and cancer prevention.^15^ On the history of surgery as a method of cancer prevention, Ilana Löwy’s work shows that, in the early twentieth century in the United States, Britain and France, surgeons came to believe that removing precancerous lesions averted the danger of malignancy. This prophylactic surgical practice later led to surgical interventions performed on women with a hereditary predisposition to cancer (breast, ovarian and cervical) even though they were healthy.^16^ A similar risk perception arose in Taiwan in the notion of the useless and pathological uterus. My analysis shows that, as an instance of the history of the globalised medical practices surrounding cancer prevention, the embodied risk of uterine cancer was set at an earlier point than the stage of pre-cancer, and it did not require any genetic testing. Furthermore, women’s bodies were portrayed as inherently more complex and pathological than men’s bodies, as seen, for instance, in the coverage of gynaecological diseases and birth control in the popular health literature in Taiwan. The health manuals for a popular audience reveal that the popularization of cancer risks went hand-in-hand with the ob/gyn’s emphasis on surgery.^17^

The American influence on medicine in Taiwan during the Cold War was extensive, ranging from health policy, nursing and medical education, medical administration, medical practice and family planning campaigns. American recommendations played a vital role in shaping the long-term health planning of Taiwan, as neither a national health policy nor a central health organization existed before 1971.^18^ Medical education in Taiwan was going through a transformation at the time, from Japanese colonial medicine to American standard medicine,^19^ as was nursing education and the nursing profession.^20^ In medicine, not only did a large number of Taiwan’s new medical graduates go to the United States for career advancement, ^21^ but, with USAID (United States Agency for International Development) support, other medical professionals, including ob/gyns with practices in Taiwan, also went to the United States for training.^22^

Modern obstetrics and gynaecology in Taiwan established itself based on a surgical orientation, and hysterectomy, abortion, tubal ligation and caesarean section are major skills of the trade. During the 1950s, ob/gyns learned from their mentors – trained in the colonial era – the valued technique of radical hysterectomy for the treatment of cervical cancer.^23^ Abortion (dilation and curettage) was also a common procedure in ob/gyn clinics from the 1950s, even though abortion was illegal until 1984. Tracing the history of caesarean sections in Taiwan, Daiwie Fu (Da-Wei Fu) points out that, in the early 1970s, VABC (virginal birth after caesarean section) was abandoned in favour of serial C-sections.^24^ Articles on tubal ligation and surgical treatments for cervical cancer featured prominently in the official journal of the Association of Obstetrics and Gynecology of the Republic of China (now Taiwan Association of Obstetrics and Gynaecology), *Journal of Obstetrics and Gynecology of the Republic of China* (now *Taiwan Journal of Obstetrics and Gynecology)*, launched in 1962. Looking back on their careers, ob/gyns often took pride in the large number of births, surgeries and pap smears they had performed in their career.^25^ Obstetrician/gynaecologists’ surgical skills were instrumental in both family planning campaigns and cervical cancer prevention; the former included abortion and installations of thousands of Loops. When the cancer prevention campaign (focused mainly on cervical cancer) began in the 1970s, they again took part in cancer screening.^26^ By offering reliable birth control and disease prevention, their surgical orientation grew alongside both the promotion of family planning and the cervical cancer prevention campaign.

The history of wuyong in Taiwan resides in the intersected history of family planning and cancer prevention. Recently there has been a growing body of literature on family planning in East Asian contexts, and women’s health is a critical issue. Yu-Ling Huang notes how population control, particularly data produced by fertility studies, helped shift the focus of population control to the reproductive behaviour of women. But the ways in which competing methods were rationalized and might have affected how women’s reproductive body, particularly the uterus, was perceived remains unexplored.^27^ In the case of South Korea, John DiMoia points out that population control comprised control of both number and quality of population and that both types of control had consequences on women’s bodies, and in a later chapter he also shows how Korean masculinities were reshaped by the state in order to convince men to accept vasectomy.^28^ This paper builds on this work on the history of family planning in East Asia to understand its ramifications on women’s health, an aspect that has not yet been thoroughly explored.^29^

In the case of how the uterus became useless and pathological, birth control was not the only issue at work; the emergence of cancer prevention, particularly cervical cancer, was also critical. These two subject areas have been treated separately in the scholarly literature, and, moreover, much of the work on the history of cancer has been centred on the West.^30^ By showing the joint influence of population control and cancer prevention on the ways in which the uterus became a bio-political object significantly mediated by surgery, this article contributes new insights into the historiography of both fields. In particular, I hope to join the projects in exploring East Asian bio-politics, or, as Francesca Bray puts it, the theme of ‘complex meshing of biology, body, and citizen that underpins projects of biological nation building and molds the forms of modern subjectivity’.^31^ In what follows, I will discuss these three aspects of the history – that is, surgery, family planning and cancer prevention – to elucidate the history of the making of a useless and pathological uterus. First, I trace the early use of surgical method (tubal ligation) before family planning, and then I describe how this surgical orientation developed along with a rational mindset of reproduction in the context of family planning. This rational mindset, expressed through the ideas of ‘useless’ and yilao yongyi, was a dramatic departure from a rural past that had valued fertility. Then, I describe the history of the cervical cancer prevention campaign to show the context in which the notion of a pathological uterus took root. The analogy made between the uterus and appendix was particularly revealing of this sense of disease risk.

## A surgical approach to birth control: before and after family planning

The desire to limit birth had been in existence since, at least, the early twentieth century in Taiwan.^32^ Before the arrival of family planning in the mid-1960s, upper- and middle-class women – teachers, writers, professionals and so forth – were early adopters of birth control, as indicated in women’s own accounts.^33^ Several methods were available to women, including the Ota ring (introduced from Japan in the 1930s),^34^ condoms, spermicide and rhythm and withdrawal methods, but these provided no guarantees. Condoms and spermicides were available mostly at pharmacies, while ob/gyn clinics provided knowledge of the rhythm method and performed tubal ligation (a method in which the fallopian tubes are severed and sealed or ‘pinched shut’) and abortion procedures.^35^ Even though tubal ligation was probably used mainly by elite women, it stood out as medical progress to liberate women from the on-going vigilance that many of the methods required.

As early as the 1930s, a small group of upper- and middle-class women had already been using tubal ligation as a contraceptive method before the official family planning project began.^36^ Shuang-Sui Lin (1901-1968), the wife of Tsung-Ming Tu (Cong-Ming Du) (1893–1986), one of the most prominent physicians in modern Taiwan, used tubal ligation as a method of birth control after she had given birth to five children. As Tu’s daughter, Shuchun Tu, recalled in an oral history interview: ‘At that time this [tubal ligation] showed progressive thinking, as very few people would get this done’.^37^ Similarly, midwives, a group also at the forefront of modernity since the colonial period,^38^ were likely to support and have tubal ligation. Cai Shuang Dai (b. 1911), a midwife, who began her practice in 1934, had a tubal ligation after she gave birth to her fourth child in 1949. She later suggested to her younger brothers’ daughters-in-law that they do the same.^39^ When his wife had a tubal ligation at the time of an abortion in 1950, the prominent physician Xin-Rong Wu (1907–1967) wrote in his dairy, ‘We have decided to utilise the highest science to regulate we humans’ natural life’.^40^

Even though sterilization by tubal ligation was used, the uterus was not yet necessarily the target, in part, because both the technological and material means for performing hysterectomy and C-section were not available. It was not until the early 1970s that major surgery such as C-section became relatively safe; for instance, the mortality rate for C-section at National Taiwan University Hospital had gone down from 1.2% in 1951 to 0.3% in 1971.^41^ Likewise, before the 1970s, hysterectomy for birth control was considered excessive. In 1951, a worried man wrote to the Reader’s Service Column of *Lianhe Bao*:

‘I live in the countryside… Now my wife is pregnant again, and we both agree that…we should not have any more, as we already have four children… I would like to ask…if we should do “shou-shu [surgery],” such as cutting the uterus or tubal ligation?’

To answer this man’s worry, the expert recommended tubal ligation over hysterectomy: ‘If you want surgery to be done, tubal ligation is enough; no need to cut out the uterus’.^42^

The family planning project officially began in 1964, and the surgical approach and reproductive rationality would expand to include women in the rural areas. It first deployed field workers to educate married women on limiting births, used mass media to disseminate propaganda, coordinated elementary schools asking children to bring home informational cards and pamphlets for their mothers, and conducted several well-known studies on effective means of carrying out these various efforts.^43^ It reached deep into the rural population. After the 1970s, the family planning literature also reached beyond married women to target high school students. In 1973, as part of the expanded family planning that was incorporated into the Six-Year Economic Construction Planning, the Department of Health distributed 1 400 000 copies of *Weiyu Choumou* [Saving for a rainy day],^44^ a pamphlet containing information about family planning, to junior, senior and vocational high school students, as well as junior college students. In 1975, the senior high school curriculum began to incorporate family planning, and local census offices handed out *Xinhun Jiating Jihua Shouce* [Family Planning Manual for the Newly Married]^45^ to couples at marriage registration.

One of the campaign’s long-lasting achievements was the popularization of reproductive rationality, the idea that women can be rational individuals who see the advantage to family welfare of having fewer children than average, which promised less burden for parents and more education for children. The recommended number of children went down from the 1960s to the 1970s. In 1967, Family Planning promoted ‘Wusan’ [five three’s, 33333] of having one’s first child after 3 years of marriage, having the second one after another 3 years, having no more than three children and completing all births before the age of 33. In 1969, it became ‘Zinu shao Xingfu duo’ (fewer children more happiness), and, by 1971, ‘3321’ (one’s first child after 3 years of marriage, the second after another 3 years, two are perfect and one is not too few). A downward trend can also be seen in the number of children women perceived to be ideal. In 1965, it was, on average, 3.96, including 2.30 sons; by 1980, among 34% of women surveyed, the ideal number of children was 2 (sex of children was not indicated). The birth rate was 4.825 in 1965 and it dropped to 1.885 in 1985.^46^

The family planning campaign made various contraceptive techniques available to married couples, and initially surgical methods did not dominate, despite the fact that Tz-Chiu Hsu (Zi-Qiu Xu), the head of the then National Health Bureau, observed that the most commonly used contraceptive methods were abortion and tubal ligation.^47^ In the period between 1964 and 1976, IUDs, rings and Lippes Loops were the number one method (64%).^48^ Judging by the fact that Loops were cheaper than rings (80NT for a ring, 30NT for a Loop) or even free if installed at a public facility such as public hospitals and health stations, Loops were likely more common. The Lippes Loop, a type of IUD that had just been invented in the US, was introduced in 1962 after a study conducted in that year (Taichung Study) had found that the Loop had a high acceptance rate in Taiwan.

Why did the Loop become the major method? As mentioned earlier, the U.S.-led family planning in Taiwan heavily promoted IUDs.^49^ The family planning campaign framed the Pill as a method mainly suitable for newly married couples not yet ready to raise children, which means it was seen as a temporary solution. In addition, women had many reservations about taking the pill. Women’s letters of inquiry to the popular magazine, *Fengnian* (Harvest), pointed to a number of problems, including ‘I have been taking the pill and feeling nausea, is it normal?’ ‘I forgot to take it, what should I do?’ ‘If I were to take it long term, would it harm my health?’ ‘Would it cause fetal abnormalities in the future?’ ‘Does it cause cancer?’ ‘Can I take the pill and still have my ginseng chicken?’^50^ Even though the answers sought to reassure the reader of the Pill’s safety, these questions nonetheless reflected women’s reservations and suspicions. Indeed, a family planning worker’s analysis of why the pill was not well-received included side-effects, lack of knowledge, newspapers’ scepticism, and having to take it every day.^51^

Unlike South Korea where vasectomy was promoted heavily by the state,^52^ the vasectomy rate was low in Taiwan, at only 0.24% among all contraceptive methods in 1964, indicating an uneven distribution of birth control burdens by gender. In the 1970s, tubal ligations outnumbered vasectomies by 10–0.8.^53^ There are several reasons why this was the case. Certain popular perceptions that the family planning campaign was eager to dispel might explain the low acceptance rate. Popular articles frequently emphasized that vasectomy was not castration and, therefore, would not compromise a man’s masculinity and would not be harmful to the body, but these were nonetheless palpable concerns.^54^ A man who underwent vasectomy might also be considered un-filial–so much so that men would do it clandestinely before the 1960s. Bin-Yu Huang, writing in the 1970s about her husband’s vasectomy two decades earlier, praised his determination and action to undergo the surgery. He had to take a three-day trip to Taipei to do it, and he told their neighbours that he had had an appendectomy.^55^

In fact, the family planning campaign soon promoted the Loop exclusively.^56^ In part because the rural population, where the birth rate was still relatively high, was thought by health planners to be ignorant and, therefore, not amenable to methods that required knowledge, training or self-discipline; for instance, it was thought that they could not be relied on to recognise the menstrual ‘safe’ period, take the Pill or use condoms.^57^ Even though Taiwanese medical authorities claimed that the Loop was especially suitable for Taiwanese women,^58^ it caused a significant percentage of adverse reactions, including spotting, bleeding, dysmenorrhea, lumbago, lower abdominal pain and perforations of the uterus.^59^ This had an unintended consequence: wanting to avoid such complications and desiring a more guaranteed solution, many women found a surgical approach to birth control appealing.^60^ Installing the Loop was only a step away from sterilization. For those women who had bad reactions to the Lippes Loop, the government encouraged voluntary sterilisation (tubal ligation) by providing financial compensation, and beginning in 1979 it was free in Taipei. As a result, the percentage of sterilisations rose precipitously from 1977, when it was 14.52% (109 722/755 465), to 1990, by which time it had climbed to 40.33% (656 680/1 628 254).^61^

The ob/gyn profession also aggressively promoted tubal ligation and the idea of yilao yongyi (one effort, once and for all). The population expert, Dong-Ming Li, wrote that, ‘When a couple have had the ideal number of children and have determined not to have any more children, in order to avoid getting pregnant again, surgical method is the simplest, safest, and most effective method’.^62^ In the mid-1970s, when Dr Shih-Chu Ho (Shi-Zhu He) (b. 1946) was a resident at Taipei Veterans General Hospital, she recalled, if the woman was over 28 and had already given birth to two children, the chief resident would ask her during rounds: ‘Did you ask her to sign the consent form for tubal ligation yet’? If answered negatively, ‘it would seem that I had not fulfilled my responsibility as a resident’.^63^ Writing for the popular magazine, *Jiankang Shijie,* Dr Zi-Yao Li (1927–2015), a prominent ob/gyn of National Taiwan University, declared that tubal ligation was the best contraceptive method because it was a yilao yongyi method.^64^ Similarly, a study carried out by Chien-Dai Chiang (Qian-Dai Jiang), Chang-E Xu and Pei-Hua Wu in the early 1980s concluded that tubal ligation was the most appropriate method for family planning and should be promoted more.^65^

Yilao yongyi was a phrase frequently used by both physicians and women, and it even appeared as a key term in a survey on women’s motivations for sterilisation. Yilao yongyi simultaneously spoke to women’s determination to not have more children and to their perception of convenience since their ob/gyns promised that, unlike other methods, it was a one-time solution.^66^ This strong sense of rationality in the decision-making around tubal ligation is also seen in its timing; according to the aforementioned study by Chiang, the majority (89.23%) of tubal ligations accompanied other medical procedures, such as right after giving birth (whether vaginal or caesarean), abortion or other gynaecological surgeries.^67^

The appendix was similarly often removed during open abdomen surgery, such as C-section or hysterectomy, and, to promote tubal ligation (via abdomen), the family planning literature highlighted appendectomy as an additional benefit to tubal ligation. The free pamphlets distributed widely by the family planning project promoted it as ‘[a surgery that] only requires poking a small hole. It is simple and safe, and one can do appendectomy at the same time’.^68^ Promoting tubal ligation in this way, the family planning project literature asserted that the uterus and the appendix were similarly dispensable organs. Indeed, appendectomy had direct associations with hysterectomy since the former was often a ‘bonus’ procedure during surgery.^69^ National Taiwan University ob/gyn Dr Zi-Yao Li (1927–2015) published an article in 1976 suggesting that ob/gyns cut more appendixes than general surgeons since the former would cut the appendix ‘incidentally’ whenever they opened the abdomen.^70^ Hai-Tao Zhao, in an account of her career as a nurse, told the story of a surgeon who, upon adding an appendectomy to a patient’s emergency hysterectomy, said: ‘To carry the good deed through, I will cut off her appendix too, so that she will not have any trouble in the future’.^71^

How the appendix came to be seen as a useless and risky body part is a subject beyond the scope of this paper. Briefly, it emerged in a social and historical process tying disease to occupation. Appendectomy had been a top surgical procedure since at least the late 1950s.^72^ By the 1960s, it had become a way of managing the risk of appendicitis, and many took the precaution of removing it, most commonly sailors, before long periods at sea. Dong-Hui Gao (1932–2012), a surgeon whose practice flourished during the 1960s–1980s in Hengchun, a small town near the ocean, remembered that when he first started his practice, ocean fishing was prosperous and many farmers went into sailing. He would regularly perform prophylactic appendectomy on sailors, sometimes more than ten cases in a day.^73^

Therefore, when the ob/gyn Xin-Xing Zhang compared the uterus to the appendix in the 1970s, portraying both as useless and potentially life-threatening, it was a familiar idea:

‘If a woman has followed her own family planning and does not want to become pregnant again, it is not necessary to keep the uterus in the body. Its existence is like Mangchang (appendix), a kind of appendage’.^74^

Indeed, ‘wanting to be sterilised’ had become one of the indications for hysterectomy, as listed in Dr Zhong-Xiu Ou’s essay, ‘Talking about Hysterectomy’.^75^ And this notion was popularized in health manuals such as *Xiandai Funu Baojian* [Keeping Health for Modern Women], a collection of short essays written by ‘famous physicians from all over the country’ and with the prominent ob/gyn pioneer, An-Chiun Chen (An-Jun Chen, 1931–2009), as the editor, which contains several other essays that promote prophylactic hysterectomy.^76^ In the translated popular health book, *Ni Xiang Zhidao de Aizheng Zhishi* [The Knowledge You Need to Know about Cancer], the analogy was carried further to frame the uterus the same as the appendix in its irrelevance to femininities – ‘Removing uterus is the same as removing appendix – neither will change women’s characteristics’.^77^

Hysterectomy as a method of sterilization was being practiced at least by the 1960s before it appeared in health manuals, as my oral interviews with women indicated. Women in physicians’ families were most notable. In my interview with Ms Xu (b. 1951), who worked at a women’s hospital as a nurse in the mid-1960s, she recalled that both the wife and mother-in-law of the head physician underwent hysterectomy when ‘they decided not to have any more children’. The mother-in-law was the younger sister of the city mayor, who was also a physician. The wife’s hysterectomy was done by the head of the hospital, her husband. Interestingly, during about the same time in the United States, it was noted that ‘doctors’ wives have proportionally more hysterectomies than any other group.^78^ Ms Xu noted:

Back then, doctors had this idea that if you don’t want to have any more children there is no reason to keep the thing [uterus] because it might cause trouble. The wife of the head physician had a hysterectomy soon after she had had four children… Her husband also said that being without a uterus avoided trouble… it wouldn’t cause any disease.^79^

To Ms Xu, much like the new material things that she first encountered at the ob/gyn hospital, such as sanitary pads, hysterectomy was a sign of progress – women could now rid themselves of menstruation, unwanted pregnancies and potential disease in one fell swoop. Not many women could afford hysterectomy, but the fact that women in physicians’ families were doing it from the 1960s added a sense of modernity to hysterectomy – a convincing exemplar of the rational utility of these modern invasive procedures.^80^

## Women’s big enemy

If the government’s family planning project defined the uterus as ‘the place for the fetus to grow’ and rendered it useless after enough children were born (in an era when pregnancy was still a tangible physical risk), how did the uterus come to assume the second characteristic of putting a woman at risk of cervical cancer (therefore, a threat to life), similar to the risk the appendix carried (appendicitis could be lethal)? The cultivation of such a risk emerged when several uterine conditions, including abnormal bleeding, myoma and endometriosis, were made into indicators of cervical cancer risk to be eliminated by hysterectomy. This hybridization of the discourses of reproductive rationality and disease risk turned the uterus into a useless and pathological object.

Cervical cancer had been the most common of women’s cancers in Taiwan since the second half of the twentieth century.^81^ According to a study based on 1,869 surgical and autopsy specimens by Shu Yeh (or Shu Ye, 1908–2004) and E.V. Cowdry (Yeh being a prominent pathologist at National Taiwan University) in 1954, over half the tumours among females were ‘carcinoma of the cervix uteri’ (55.97%).^82^ From the 1950s to 1970s, it was on top of the list for women’s cancers,^83^ and it commonly appeared in the diaries of the gentry class.^84^ In the period between 1973 and 1974, the largest number of cancer deaths for women was cervical cancer (686).^85^ When compared internationally, the mortality rate was high. In the late 1960s, the mortality rate for cervical cancer in Taiwan was 14.24 per 100 000, as opposed to 11.85 in Japan and 9.67 in the United States.^86^ It had been called the ‘public enemy of all women’ since the 1950s.^87^ As one of the popular health manuals states, ‘for those who are slightly advanced in age, none have not heard of it’, and it is ‘the most troublesome and horrifying disease’.^88^ Until 1997, the gynaecologist, Dr Shih-Chu Ho (Shi-Zhu He), still called it the ‘number-one enemy of Taiwanese women’.^89^

Pap smear screening began in the early 1970s, substantially lagging behind other countries’ efforts.^90^ The US began in the 1950s, and Japan adopted it in 1961.^91^ Explaining why Pap smear screening began so late in Taiwan, Daiwie Fu suggests that, in addition to a lack of government policy and funding, and women’s hesitancy to see male gynaecologists, the ob/gyn community was heavily invested in a surgical approach focused on radical hysterectomy as the pinnacle of the trade. Thus, it was difficult for ob/gyns to make the transition from a surgery- and hospital-based practice to a decentralised and community-based practice of screening.^92^ However, the family planning and cancer prevention project helped to bring the care of women closer to such a style of practice.

Before cervical cancer prevention measures became common, most of the cases of cervical cancer occurred at an advanced stage, and radical hysterectomy and radiotherapy were the main treatments. Physicians regretted that little could be done, and the quantity of available radium was often limited.^93^ As the popular saying had it, ‘wenai sebian’ (One pales with fear upon hearing cancer); the disease meant death.

Women dreaded the disease and its treatment. Man-Qing Xiao recalled her experience in her memoir:

‘I remember it was 1967…on a night when my children were all deep in sleep I wrote five letters, each to my five children. I didn’t know what the result of the surgery might be. I recalled the wife of Changrong’s colleague, Mrs Ma, who had died from cervical cancer surgery at Dr Chien-Tien Hsu (Qian-Tian Xu)’s clinic, leaving five young children behind. It’s heartbreaking. Or, like chubby Mrs Lee in neighboring Zhongshan lane, who died not long after two operations at the Veteran’s Hospital. Or, the wife of the head of the Police Branch of Taoyuan City who also had this disease and died not long after surgery’.^94^

Cervical cancer was a common occurrence in Xiao’s social network, and many had died from the disease or the surgery. She feared death and leaving her young children behind. Xiao’s account was a typical story from the time; during the 1950s–1970s, cervical cancer patients in newspaper accounts were often poor middle-aged women with many children.^95^ Some of these accounts discussed poor women committing suicide as a result of the incurable disease and life’s hardships.^96^ Nevertheless, fear of cancer was not limited to the poor. Hua Yan (Ting-Yun Yan, b. 1926), a prominent writer, conveys her experience of myomectomy and fear of cancer:

‘To be honest, I am afraid of exams. Once being examined, there is not a single cell of a healthy person that will not possibly get the most terminal diseases (Zuijue de Zheng) in the world’.^97^

Husbands wrote about their loss and heartbreak. ‘Cervical cancer took away my wife’ was a story about how Chun-Chan’s (Spring Silkworm, a pen name) wife died from cervical cancer in 1967 as a result of misdiagnosis and delayed treatments. *Jiang Gui-qin’s True Story* is a father’s memoir about his diseased daughter, which also contained a chapter about his wife who had died from cervical cancer at the age of 44 after three surgeries, leaving their daughter motherless.^98^ Physicians also felt compelled to write about the tragedy of human suffering. Dr Tian-You Lin’s memoir, ‘Lies’, describes two memorable patients in the 1960s, his elementary school classmate and the classmate’s wife. The husband was diagnosed with stomach cancer and the wife was later diagnosed with cervical cancer. The couple separately told their physicians not to disclose the other’s disease. The wife’s cancer was stage III and not operable.^99^ Physicians’ stories about women’s paralysing fear of the disease are also common in the popular literature of the era.^100^

Efforts at disease prevention were limited before the 1970s; Zhong-Quan Zhang, an ob/gyn at Taipei Municipal Zhong-Xing Hospital (currently Taipei City Hospital Zhongxing Branch), reflected that in the 1960s most of his colleagues busied themselves in treating patients and paid little attention to prevention.^101^ He did not begin conducting cervical cancer screening until the early1970s.^102^ In fact, prior to the 1960s, the only efforts made were by National Taiwan University Hospital (hereafter NTU Hospital), which began a small-scale, subsidised examination programme in the early 1960s, after establishing a special clinic for treating cervical cancer in the 1950s.^103^ As the two hospitals were in Taipei, these efforts mainly reached women in Taiwan’s largest urban area.

## Cervical Cancer Prevention and Embodied Risk

In the process of the promotion of cancer prevention and under the shadow of cancer risk, the notion of yilao yongyi was gradually applied to hysterectomy as it had been applied to tubal ligation. According to Xiang-Da Wu (1938–2018) and his colleagues, prominent ob/gyns at Taipei Veterans’ Hospital, prophylactic hysterectomy became popular in the mid-1970s. The first large-scale cancer prevention campaign began at this time, led by the Cancer Society of the Republic of China and the public health expert, Pesus Bise Chou (Bi-Se Zhou). Cervical cancer figured prominently in the gendered cancer prevention discourses and measures,^104^ and it was also a model cancer for the idea of early detection and early treatment; the campaign popularised the idea that, if it was caught early, it could be 100% cured.^105^

The campaign’s main tasks were to educate women and to make individual ob/gyn practitioners the campaign’s partners. Like the family planning campaign, it identified rural women, perceived to have no knowledge of prevention, as their main target. Public health experts and physicians alike saw rural women’s ignorance as an obstacle.^106^ The campaign sought to educate women via films, lectures, cancer survivors’ experience-sharing and small-group meetings at local farmers’ associations. Women were told to accept Pap smears and seek regular follow-up surveillance of their body; the message was to be constantly on guard and undergo gynaecological examination regularly.^107^ I will return to the problem of gynaecological examinations later.

To convince women to take preventive action, such as accepting an unpleasant Pap smear, the prevention campaign employed scare tactics and emphasized the deadly consequences of undetected and untreated disease.^108^ For example, the film, *Shengsi zhijian* (Between Life and Death), purportedly the first of its kind, was meant to ‘flash a warning light’ at the intersection of life and death ‘under the horrible shadow of cancer’.^109^ The film featured well-known actors, such as Chang Feng and Mei Fang, and, in a time when not much entertainment was available, the showing of such films was often considered the highlight of a cancer prevention event, attracting many from afar, especially those living in the remote rural areas. The campaign reported that, after watching the film, women sought free Pap smears at their local ob/gyn clinics and husbands brought their wives to the ob/gyns. As a result, clinics were busier than before.^110^

The spread of fear was double-edged.^111^ Women were motivated to action by fear; some would rather ‘overreact’ than accept ambiguous results, which would mean living with the risk. The campaign created two main groups of women – cooperative patients and non-participants (who refused to accept a Pap smear and follow-up exams). Those who were willing to cooperate ‘erroneously took the attitude that they were not far from death, which greatly troubled their minds’.^112^ If a test indicated something suspicious, a woman was encouraged to do further tests. As research has pointed out, women’s experience of cervical screening may have reinforced a sense of risk, particularly for those who received suspicious results.^113^ The leader of the campaign reported a case of ‘overreaction’ – a woman who would rather undergo hysterectomy than live with the risk:

‘[There was] a patient whose Pap smear result was class III, but the tissue biopsy indicated merely cervix erosion. She was worried and went to three different hospitals to get tissue biopsies. The results were all the same – no cancer. Normally, she should have felt happy…. She complained, ‘I’d rather have cancer and cut off my uterus so I can have quick relief’.^114^

Ambiguous test results prompted many women to accept hysterectomy as a preventive measure after being encouraged by their doctor to err on the side of caution. In the late 1970s, Ms Huang’s mother had a Pap smear with ambiguous results. It read, ‘X is suspected’. Her mother was so terrified that she became bedridden for weeks. As she greatly feared surgery, she did not have the courage to do more than worry, but several of her friends who had received similar reports ‘bravely’ accepted hysterectomy.^115^

The campaign also recruited individual ob/gyns into their network of prevention; when the campaign conducted the first two cervical cancer mass screenings (between 1974 and 1978 and between 1979 and 1984), 661 and 569 ob/gyn clinics were involved, respectively.^116^ By 1977, the campaign had carried out over 60 000 Pap smears.^117^ In addition to sending those who were diagnosed with cervical cancer to ob/gyns who would provide the treatment of hysterectomy, the campaign invited ob/gyns to hold funded free clinics and offered updated cancer knowledge from the United States, such as educational films sent by the American Cancer Society.

Ob/gyns and their practice, along with popular literature (mostly written by ob/gyns such as the aforementioned *Xiandai Funu Baojian*), became part of the infrastructure for the cultivation of risk awareness. In an article meant to encourage women to receive regular exams, Tao-Sun Wang, MD, who also participated in Pap smear screening in the early 1970s, wrote about hysterectomy as one of the treatments for cervical cancer:

‘The uterus, except for menstruation and nurturing the fetus, is not very important. If a woman has had enough children and has reached middle age, the uterus is not very useful for her. Hysterectomy would not damage her femininity’.^118^

In trying to convince women to accept hysterectomy, Dr Wang repeated the notion of wuyong and argued against the popular notion that a woman without a uterus was not a true woman.

As part of their persuasive tactics, physicians might also bring up the additional benefit of insurance compensation to their women patients. For women of reproductive age having no uterus (therefore infertile) was officially compensated as a form of canfei (handicap, disability), according to several major government insurance programmes, including Labor Insurance (1950–1995), Government Employees’ Benefits and Insurance (1958–1995), and Farmer’s Health Insurance (1987–1995). Therefore, women under 45, who underwent hysterectomy, were qualified to receive handicap (later disability) compensation, regardless of whether or not they had received prophylactic hysterectomy because they had fulfilled their maternal duty.

An additional aspect potentially enforcing the notion of the useless and pathological uterus was women’s concern of modesty. Pap smears or regular check-ups were the two main weapons against cancer. However, both were resisted because the ways in which they were carried out made women uncomfortable. Women’s concern for modesty in ob/gyn exams has a long history, noted by Western-trained ob/gyns struggling to establish their practice in the colonial period.^119^ This problem continued after the Second World War and into the post-war period. Reports about the embarrassing ob/gyn visit were common. Family planning workers reported conservative women’s refusal to let men doctors install the Loop. Sometimes they asked the nurse to talk to the patient behind a curtain while the doctor worked silently also behind the curtain, obscuring the identity of the Loop installer.^120^ Women asked about clinics that had women physicians installing the Loop, and the names of such clinics were listed in the popular magazine, *Fengnian.* In addition, the exam space itself contributed to women’s resistance. In 1977, a group of physicians acknowledged that exam rooms were inadequately set up – the space was poorly regulated, people other than doctors and nurses were allowed to walk in and out of the room freely and women frequently felt embarrassed.^121^

Hysterectomy became a way to avoid this kind of risk surveillance; violation of modesty or privacy was obviated by the hysterectomy as yilao yongyi, ending the need for routine gynaecological examinations. When Ms Miao Young was being discharged from the hospital after her hysterectomy (due to myoma), she was given a business card by her gynaecologist, who commented: ‘You have *biye* (graduated) from here [ob/gyn department]’, implying that she was done with ob/gyn clinics and could pass the business card to other women who might want to ‘graduate’ from the ob/gyn department. Ms Young’s story suggests that having a uterus meant a woman would never be free from uncomfortable ob/gyn visits. Similarly, Mrs Hong explained, ‘[after hysterectomy] one will not have to take off one’s pants and expose oneself to others’. Ms Xu mentioned that many of her friends in the Buddhist community, especially the nuns, did not think it was respectable to expose themselves to an ob/gyn regularly, and therefore they underwent hysterectomy.

In addition to the cancer prevention campaign, another important place to observe how medical knowledge portrayed women’s body as a site of cancer risk was a popular health literature for women, a genre that emerged in the late 1960s and early 1970s and included health manuals and newspaper columns. Popular health manuals tended to pathologise the female body, and, in this light, cervical cancer and its early symptoms were featured in most of the health manuals.^122^ In the 1950s, the discourse of cancer risk had already penetrated the existing genres: both popular health literature and traditional Chinese medicine. Ob/gyns had been warning women in the news media that baidai (‘whites strips’, whites, leucorrhoea) were a sign of uterine cancer.^123^ The popular literature of traditional Chinese medicine manufacturers also appropriated Western medical knowledge on cancer and emphasized the importance of menstrual regulation, offering remedies for menstrual problems and whites.^124^ However, at this point, neither advocated hysterectomy as a form of prevention.

The 1970s saw a marked increase in the number of news reports, magazine articles and health manuals for women on cervical cancer, which, together with the cancer prevention campaign, contributed to the cultivation of risk.^125^ Specifically, women were told to watch out for two warning signs of cervical cancer, bu zhengchang chuxie (abnormal, irregular bleeding) as well as whites, in addition to conditions, such as myoma and cervical erosion, which were also linked to cancer. Abnormal bleeding was a frequent key term in the popular writing, and women were educated to seek doctor’s help upon observing it; signs of bleeding now pointed to the problem of the uterus. Sumama (b. 1933) visited the ob/gyn clinic in her neighbourhood for ‘abnormal’ bleeding around 1980. Without doing any exams, the doctor simply told her that something bad was growing inside her uterus and a hysterectomy should be done. She decided to get a second opinion, however, and it turned out that the bleeding was a sign of menopause.^126^

Another salient example of how a symptom was seen differently once the risk discourse spread was myoma, which has been the number one indication for hysterectomy since the 1970s.^127^ The Chinese term for myoma, jiliu, is a term that can be easily confused with cancer, zhongliu, as they share the word liu (tumour). During the 1960s, ob/gyns’ recommendations regarding myoma were conditioned by women’s marital status and age. In 1967, an article, ‘Why conduct hysterectomy’, published in the popular health magazine, *Dazhong Yixue*, listed four conditions that would require hysterectomy: tumour (both malignant and benign), infection (especially, uterine dysfunction and haemorrhage) and uterine prolapse due to endometriosis or tubo-ovarian abscesses. Compared to the case of malignant tumours, in which the removal of the uterus, ovaries and fallopian tubes should be carried out hao-wu-bao-liu (without reservation, or leaving nothing), in the case of benign tumours, the author writes that it depends on the patient’s age; ‘If the patient is over 40, has many children, and has had severe anemia’, she should have a total hysterectomy. If the patient is young, has not given birth yet, and the uterus is still healthy, she might consider partial hysterectomy to keep her married (sex) life unaffected.^128^ In other words, risk was evaluated differently depending on a woman’s marital status, reproductive status and age.

However, health experts gradually began to write about the potential for myoma to become something malignant. Obstetrician/gynaecologists in their practice expressed the same concern. Women often heard and worried that ‘something bad might be growing inside the uterus’.^129^ For example, Mrs Lin (b. 1953), a dressmaker, had tubal ligation in the early 1970s. Almost 20 years later, in 1991, Mrs Lin went to her local ob/gyn for an ultrasound check-up, as many of her customers had recently been diagnosed with cancer. The doctor suggested that the tubes be removed, but he asked permission from her family to do a hysterectomy during operation because he thought the uterus was not something to keep.^130^

Other uterine diseases also contributed to the problematizing of the uterus. For instance, the case of endometriosis, a disease whose numbers increased dramatically after the introduction of laparoscopy technology. Impossible to cure but rarely fatal, it acquired the nickname of ‘benign cancer’, an oxymoron that still indicated cause for concern. Again, the treatment options were highly dependent on the woman’s marital and reproductive status. If she was young and not married, the doctor would recommend marriage, under the assumption that she should have children as soon as possible. If she was married without children, the doctor would treat her (assumed) infertility. If she was married with enough children, a hysterectomy would be recommended.^131^

Some physicians went so far as to advocate the notion that having no symptom for cervical cancer was in itself a symptom. Citing a Japanese authority, Dr Zhang wrote,

‘We should accept Kushima’s suggestion that gynecology textbooks should list no symptom as one of the first, early symptoms of cervical cancer to increase women’s awareness. Since early cervical cancer is not symptomatic, regular examinations are a necessary prevention measure’.^132^

Dr Zhang was not alone in this view; the ‘no symptom as the symptom’ can also be found in the popular literature.^133^

Almost every woman interviewed in this study had heard from her doctor that after giving birth the uterus was ‘useless’, and if you leave it alone, it might develop into something evil.^134^ Their physicians’ common refrain was along the lines of: ‘You do not know what might be growing inside [the uterus]’, which were the specific, ominous words of Ms Zheng’s doctor. The uterus’s interior was a dark mystery, a source of fear stoked by their physicians’ statements. Other interviewees reported hearing or using a related expression: ‘The uterus is not something you want to keep’ (Lin, Xu, Mrs Wang, Ms Miao Young’s doctors and Ms Miao Young). Hysterectomy was considered a logical step in response to cancer fears.

Not surprisingly, some women demanded hysterectomies from their physicians out of fear. For example, Ms Miao (b. 1952), who worked at a textile factory, after giving birth to three children, feared getting pregnant again. She had had two abortions by the age of 30. If it was fear of unwanted pregnancy alone, a tubal ligation would have sufficed. But she was also troubled by the volume of her leucorrhoea, which made her worry about cervical cancer. In 1982, she demanded the local gynaecologist remove her uterus, but the doctor was hesitant to do the surgery, saying ‘you are too young’. She nevertheless persisted and eventually convinced him to do it. She had heard from her fellow women workers that having too much whites was a sign of cancer. While she did not see working in a factory as a hardship, she certainly did not want to have any more children or get cancer. Dr Yi-Hung Zhan (Yi-Hong Zhan) writes about a 23-year-old woman who similarly requested hysterectomy to avoid ‘houhuan’ (future troubles), and another 39-year-old woman who, troubled by leucorrhoea and fearing that it might be cancer, also made the request. He cautioned that, even though the surgery was relatively safe, there were potential side-effects, such as infection and damage to the bladder and urethra.^135^

More often than not, newspaper advice columns promoted the risk posed by the uterus and of not having a hysterectomy, and the sources of information were often from the US and, to a lesser degree, Japan, both of which carried some degree of imperial/social authority. The following, attributed to the American Cancer Society and appearing in translation, is just one example, in which every organ in a woman’s reproductive system was depicted as potentially lethal.

‘If a woman has had total hysterectomy (including uterus and cervix), of course, she will be free from the danger, but if the hysterectomy is not total, she will still have the risk of cervical cancer. Likewise, if the ovaries are not removed, they might become the prime source of disaster’.^136^

Another example, also a translation into Chinese from an American source:

‘According to Dr Lu Ka Si, a woman over 40 when facing hysterectomy should also remove her ovaries to prevent uterine and ovarian cancers…Therefore, Dr Lu Ka Si recommends that all women older than 40 should remove their ovaries’.^137^

**Figure 1:**
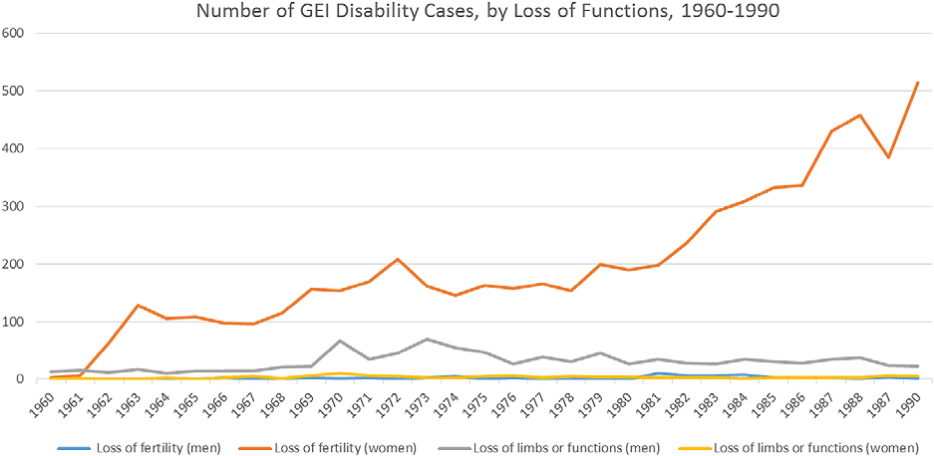
Source: Central Trust of China [Taiwan], *Statistical Data for Government Employees’ Insurance Republic of China,* (1991): 226–227.

It is in this context of heightened sense of cancer risk that the notion of a pathological uterus emerged.^138^ The pathologisation of the uterus was a critical element in the transformation of hysterectomy from a treatment to a prophylaxis. It is difficult to get an exact picture of the extent to which the practice of hysterectomy increased, since comprehensive data for medical procedures were not available before the implementation of National Health Insurance in 1995. However, we may extrapolate from statistics of particular groups of the population, mainly those who were enrolled in Government Employees’ Insurance (since 1958), since it paid cash benefits for loss of fertility.^139^ According to Statistical Data for Government Employees’ Insurance (GEI), the number of claims for ‘loss of fertility function’ for women steadily increased from 3 in 1960 to 514 in 1990, the cumulative number being 6,233.^140^ Moreover, since the mid-1960s, removal of the uterus was at the top of the list for ‘disability’ cases, and by 1990 it had reached 71.8% (514 cases) of all cases in the Loss of Fertility category.^141^ In contrast, the number for men under the same category remained constant over the years, the cumulative number reaching a mere 65 (Figure 1).^142^ Since its implementation in 1950, Labour Insurance has also provided cash benefits if one becomes canfei (handicapped, disabled), including loss of fertility.^143^ There are, however, no data specifically on the loss of fertility in the category of ‘Disability Benefit’. Even though the population of public employees was relatively small, its status as a stable middle-class group can be said to make it representative of a large portion of the rapidly economically developing society’s population.

Although dissenting voices within Taiwan’s ob/gyn profession were rare, public controversies over hysterectomy and other surgeries resulted in broader critiques of the medical profession. A few popular essays, appearing in translation, warned women not to accept hysterectomy too easily. For example, a translated essay from the American women’s magazine McCall’s, written by a Dr William A. Kolem,^144^ suggested that seeing the uterus as a dispensable organ and cutting it without much consideration was an extremely arbitrary decision. One of the few ob/gyns who voiced a moderately different opinion in Taiwan was the prominent ob/gyn, Xiang-Da Wu, who was also one of the translators of the feminist classic, *Our Bodies Ourselves.* Wu was weary of his fellow ob/gyns, who were acting like the surgeons who had made cutting open the stomach a ‘trend’; hysterectomy had become ‘known by all ages’, he lamented. He listed 10 indications for hysterectomy, and he particularly focused on myoma, as it was the most common indication for hysterectomies. He explained, one might consider hysterectomy only if the myoma was growing on the muscle of the uterus and extended into the inside of the uterus, as big as a 12-week foetus, causing aches, and bleeding after menopause.^145^ In professional writings, Wu’s position against unnecessary hysterectomy was more explicit. In a co-authored article on hysterectomy, noting its popularity, Wu and his colleagues concluded that ‘it is not reasonable to do hysterectomy in order to sterilise, prevent cancer, or avoid symptoms of menopause’.^146^

The fact that hysterectomy, along with other surgeries, had become so common no doubt also raised suspicions over surgical abuse. A 1977 newspaper article about unnecessary surgeries indicated that stomach, thyroid and uterus were the most frequently removed organs.^147^ An ob/gyn, Dr Zu-Miao Zhao, went so far as to publish two books entitled *Choulou de Yisheng* (The Ugly Doctors), which exposed numerous forms of medical misconduct.^148^ Bo Yang (1920–2008), a well-known writer and cultural critic, sarcastically suggested that a Kangzai Weiyuanhui (Resisting Butchery Council) be established in order to curb such a trend.^149^

Yet, the notion of the veteran uterus as useless and pathological persisted up to the end of the twentieth century. Nuquan Hui’s (Taiwan Association for the Promotion of Women’s Rights) Women’s Health Support Service Phone Line reported receiving 1 021 phone calls in the period between October 1998 and July 1999, and they found that nearly 90% of the women who went to the doctor because of myoma had been encouraged to have hysterectomy. The reasons given by the doctors were: ‘You are not going to have any more children anyway’, ‘It will save you a lot of trouble in the future’, and, ‘It’s cancer prevention’. Some of the doctors even recommended the removal of the ovaries altogether as a prevention measure.^150^

## Conclusion

As a justification for preventive measures to combat unwanted situations, the notion of yilao yongyi (one effort, once and for all) was first attached to appendectomy, tubal ligation, and, eventually, to hysterectomy in a historical process of making the uterus useless and pathological. Even though limiting births and dreading cancer were not new for post-colonial Taiwan, the response had its roots in surgical prowess that dated back to the Japanese colonial period and flourished in the American-dominated family planning and cancer prevention. The three forces formed a powerful push towards a more rational technological control of reproduction and disease.

Yilao yongyi was a history of various competing yet connecting birth control methods, as in the case of Lippes Loop and tubal ligation. Even though the Loop was heavily promoted by the United States, the later, a surgery that was already mature locally, came to be the yilao yongyi method. Women would be rid of any further work after tubal ligation. Yet, tubal ligation was not the right method if one also wanted to be yilao yongyi with cervical cancer; hysterectomy served the dual purposes of birth control and cancer prevention. The uterus, an organ that can breed lives and grow cancer, became the target to be removed. Hysterectomy was carried out as a prophylactic strike for women who were thought in need of birth control and cancer prevention.

The discourse of wuyong ‘useless and pathological’ uterus and various associated practices were a form of bio-power at work, and it involved women, surgeons, public health nurses and the state. However, in a society where women took on the sole burden of birth control, the different actors did not share the stakes equally. If women wanted to retire from maternal social duty and they could find no satisfactory birth control method, the idea that they might be better off without a uterus was appealing. One wonders what would have happened if women had not had to take on the main burden of birth control, the project of family planning had devoted more attention to male methods, and the medical professions had been less surgery-oriented. Would the uterus still have been at the centre of family planning or cancer risk discourse?

Without the cultivation of a rational reproductive mindset, as seen in the notion of technocratic ‘planning’, the uterus as useless would have been incomprehensible. Moreover, without the cultivation of a visceral sense of cancer risk, many women would not have gone to local ob/gyn clinics and the uterus would not have become a prime suspect of cancer (as opposed to being an interconnected part of the body). All of the behaviours and conditions of the uterus, including bleeding, excreting, eroding and developing myoma, were made into components of the pathologisation of the uterus. In the cancer prevention discourse, early detection and treatment necessitated regular surveillance of their bodies, and women’s aversion to gynaecological exams, combined with the fear of cancer, further rendered the uterus a problem.

In Taiwan, the history of reproductive technologies and the history of cervical cancer crossed at the nexus of hysterectomy, a practice that went from being a treatment for cervical cancer and obstetrical emergencies to a birth control method and routine prophylactic measure. The timing of action was moved to a very early point in life – that is, when the woman had had ‘enough’ children – as early detection came to be deemed inadequate in the face of heightened risk perceptions or unacceptable surveillance measures.^151^ The uterus itself was the risk, giving but also taking life. In short, women were advised to be on guard for their life as soon as they had brought ‘enough’ lives into the world, and to seek convenience by seemingly rational, techno-scientific means. Thus, the history of the uterus being *wuyong* is also the history of how yilao yongyi became desirable and hysterectomy became a reasonable solution. Yilao yongyi (one effort, once and for all) gradually adhered to hysterectomy as a convenient and rational response to having a supposedly useless and pathological organ, authorised by biomedical authority, popular health discourses and women’s testimonials.

This paper not only adds to the current literature of bio-politics in East Asian context, but by bringing the history of the useless and pathological uterus into the story, it also answers Warwick Anderson’s call to pay more attention to the ‘more intimate and private parts of public health’.^152^ The uterus was a woman’s private body part, but it was also at the nexus of colonial surgical legacy, American dominance and local medical culture.

Scholars have questioned the common assumption that biomedicine has been an unproblematic force for women’s liberation.^153^ Indeed, the construction of the useless and pathological uterus was armed with the rhetoric of progress and liberation; medicine’s progress was meant to bring liberation for women from unwanted pregnancies and cancer. Nevertheless, the history of wuyong is a case of the bio-politics of population and disease control that shaped anatomo-politics by singling out the uterus as an organ that could be removed to manage risk. The isolated uterus is very different from the view from Chinese medicine, in which the uterus is intimately connected with other internal organs (and the removal would disconnect the flow of the *qi*).

It is striking that the ways in which Taiwanese obstetrician/gynaecologists portrayed the uterus were very similar to their American and Japanese counterparts’ depictions of a useless, dangerous and troublesome organ. No substantive research is yet available on how such a notion or practice has travelled globally. However, from the fact that ob/gyns in the second half of the twentieth century in Taiwan actively promoted the ‘useless and pathological uterus’ in ways socio-culturally significant in Taiwan while engaging in international medical networks,^154^ we can glimpse how medical practice and knowledge circulated globally and, at the same time, developed local variations.

## Footnotes

1 In this article, existing English translations of Chinese names and book and article titles are used where possible, and, if a Chinese name is not spelled in pinyin, the pinyin spelling is provided in parenthesis. If no English version exists for a reference, the translation is provided by the author.

2 Xian-Jie Meng, ‘Cervical Cancer I’, *Funu Zazhi* [Women’s Magazine], July (1971), 66–7.

3

Chueh Chang (Jue Zhang), Chu-Hui Chang (Ju-Hui Zhang), Yow-Hwey Hu (You-Hui Hu), ‘Physician-Patients Communication Reported by Women of Hysterectomy’, Zhonghua Gonggong Weisheng Zazhi [*Chinese Journal of Public Health*], 16, 1 (1997), 1.

4

Ralph C. Wright , ‘Hysterectomy: Past, Present, and Future’, Obstetrics & Gynecology, 33, 4 (1969), 560–3.5776112

5

Lenn E. Goodman and Madeleine J. Goodman , ‘Prevention—How Misuse of a Concept Undercuts Its Worth’, Hasting Center Report, 16, 2 (1986), 26–38.3700076

6

Robert Pokras and Vicki G. Hufnagel , ‘Hysterectomy in the United States, 1965–84’, American Journal of Public Health, 78, 7 (1988), 852–3.338196810.2105/ajph.78.7.852PMC1350353

7 As the sociologist Anne Kasper points out, obstetrician–gynaecologists may have helped create a social context in which ‘hysterectomy is acceptable treatment for a number of equivocal indications – to avoid further pregnancies, to obviate the discomfort of menstruations, and to prevent the possibility of uterine cancer.’ Anne S. Kasper, ‘Hysterectomy as Social Process’, *Women & Health*, 10, 1 (1985), 109–28.

8 Kono Miyoko, ‘Excerpts from Our Bodies Ourselves’ [Preface of the Japanese Edition], in Sandra Buckley (ed.), *Broken Silence: Voices of Japanese Feminism* (Berkeley: University of California Press, 1997), 199–218.

9

Aldrich T. , et al., ‘Mexican Physicians’ Knowledge and Attitudes about the Human Papillomavirus and Cervical Cancer: A National Survey’, Sexually Transmitted Infections, 81, 2 (2005), 135–41.1580009110.1136/sti.2003.008557PMC1764650

10 ‘Korea’s hysterectomy rate tops OECD nations’, *Joongang Daily*, 12 June 2013.

11 Ornella Moscucci, *The Science of Woman: Gynaecology and Gender in England, 1800–1929* (Cambridge: Cambridge University Press, 1990). Ella Shohat points out that the medical discourse used the term ‘a walking pathological laboratory’ to refer to women’s bodies. Ella Shohat, ‘Laser for Ladies: Endo Discourse and the Inscription of Science’, in Paula Treichler , Lisa Cartwright , and Constance Penley (eds), The Visible Woman: Imaging Technologies, Gender, and Science (New York: New York University Press, 1998), 259.

12 The ‘uterus’ in traditional Chinese medicine is not a single organ in the biomedical sense, but refers to several gynaecological reproductive functions of the uterus, ovaries, and fallopian tubes and, moreover, is intimately connected with other internal organs. Li-De Chen (ed.), *Zhongyi Fuke Xue [Gynecology in Chinese Medicine]* (Taichung, Hongxiang Chubanshe, 2003), 27–31.

13

Daiwie Fu , Yaxiya de Xinshenti [*Assembling the New Body: Gender/Sexuality, Medicine, and Modern Taiwan*] (Taipei: Social Publishing, 2005).

14 Wen-Hua Kuo, ‘Meiyuan xia de Weisheng Zhengce: 1960 Niendai Taiwan Jiating Jihua de Tantao’ [Politicizing Family Planning and Medicalizing Reproductive Bodies: US-Backed Population Control in 1960s Taiwan], *Taiwan: A Radical Quarterly in Social Studies*, 32 (1998), 39–82.

15 Most of the work is in Chinese and on topics such as the rise of modern ob/gyn, birth control, birthing practice, abortion, pregnancy test, cesarean section, endometriosis, menarche, and health manuals. Yan-Chiou Wu (Yan-Qiu Wu), ‘Yanjian Weizhen Zhanhou Taiwan Funu Yanyunshi [Seeing is Believing: A History of Pregnancy Testing in Taiwan], 1945–1990’, *Nuxue Xuezhi* [*Journal of Women’s and Gender Studies*], 35 (2014), 1–52; Yan-Chiou Wu, ‘Bizui Zhidao: Cong Yuejing Quizeshu Yinjin kan 1970 Niandai Taiwan Duotai Shih [Evading the Law? Abortion in Taiwan during the 1970s: A Case Study of Menstrual Regulation]’, *Taiwan Shehui Yanjiu Jikan* [*Taiwan: A Radical Quarterly in Social Studies*], 45 (2012), 103–172; Chia-Ling Wu, ‘Taiwan de Xinshengzhi Keji yu Xingbie Zhengzhi [The New Reproductive Technologies and Gender Politics in Taiwan], 1950–2000’, *Taiwan Shehui Yanjiu Jikan* [*Taiwan: A Radical Quarterly in Social Studies*], 45 (2002), 1–67; Linya Tsai (Lingya Cai) and Hsiu-Yun Wang (Xiu-Yun Wang), ‘Cong Chuzhen dao Yiguankuitian: Fuqiangjing yu Zigongneimoyiweizheng de Xingqi [From Hands of Flesh to Laparoscopy: A History of Endometriosis in Taiwan], 1950s–2000s’, *Keji Yiliao yu Shehui* [*Taiwanese Journal for Studies of Science, Technology, and Medicine*], 10 (2010), 74–128. Others in English include: Daiwie Fu, ‘CS, VBAC, and an Ironic Past in Taiwan’s Obstetrics’, *Gender and Sexuality: Journal of Center for Gender Studies*, ICU, 2 (2006), 25–41; Chia-Ling Wu, ‘Have Someone Cut the Umbilical Cord: Women’s Birthing Networks Knowledge and Skills in Colonial Taiwan’, in Angela Ki Che Leung (Qi Zi Liang) (eds), *Health and Hygiene in Chinese East Asia* (Durham: Duke University Press, 2011), 160–180.

16

Ilana Löwy , Preventive Strikes: Women, Precancer, and Prophylactic Surgery (Baltimore: Johns Hopkins University Press, 2010).

17

Hsiu-Yun Wang , ‘Taitai Yixue:Taiwan Funu Yixue Baojian Chutan [Medical Knowledge for the Wife: Popular Health Manuals in Taiwan], 1950s–2000s’, Taiwan Shehui Yanjiu Jikan [*Taiwan: A Radical Quarterly in Social Studies*], 76 (2009), 91–149.

18

Tsui-Hua Yang , ‘Meiyuan dui Taiwan de Weisheng Jihua yu Yiliao Tizhi zhi Xingsu’ [USAID in the Formation of Health Planning and the Medical System in Taiwan], Jindai Shi Yanjiu Suo Jikan [*Bulletin of the Institute of Modern History*, Academia Sinica], 62, (2008), 91–139.

19 Michael Shiyung Liu, ‘From Japanese Colonial Medicine to American-Standard Medicine in Taiwan: A Case Study of the Transition in the Medical Profession and Practices in East Asia’, in Liping Bu , Darwin H. Stapleton and Ka-che Yip (eds), Science, Public Health and the State in Modern Asia (New York: Routledge, 2012), 161–76.

20

Shu-Ching Chang , ‘Meishi Huli zai Taiwan: Guoji Yuanzhu yu Daxue Huli Jiaoyu de Kaiduan [American-style Nursing in Taiwan: International Aid and the Origins of Nursing Colleges]’, Research on Women in Modern Chinese History, 18 (2010), 125–73.

21 According to a survey by Chen, during the period between 1963 and 1973, approximately 40% medical graduates went abroad, mainly to the United States, and most of them did not return. See Hung Bin Hsu, ‘Nianqing Yishi de Panghuang yu Jueze: Cong Qingxing kan Taiwan 1950–1960 Niandai Yixue Jiaoyu, Yishi Chulu ji Wailiu Wenti [Medical Education, Professional Career and the Exodus of Young Doctors in Taiwan during the 1950s and 1960s, an Analysis based on Qingxing]’, *Chung-Hsing Journal of History*, 27 (2013), 57.

22 Although no national statistics are available regarding how many physicians went to the United States for training, from the 1950s on it was very common for ob/gyns to go to the United States to learn new knowledge or technologies. Prominent figures who did this include Shi-Rong Qiu (1912–1998), Xi-Yao Chen (1922–2012), Zi-Yao Li (1927–2015), An-Chiun Chen (1931–2009), Fu-Min Chen (b. 1936) and Wen-Long Chen (b. 1941). With USAID, Shi-Rong Qiu went to Duke University to learn about radical hysterectomy in 1954. An-Chiun Chen went to Johns Hopkins Hospital in 1962, where he first learned about endometriosis. Zi-Yao Li went to Washington University to learn about hormones and infertility in 1965. Xi-Yao Chen went to New York in 1969. Fu-Min Chen went to Johns Hopkins in 1967, and he returned in 1972 and introduced the MVA (Manual Vacuum Aspirator). For National Taiwan University faculty, see Hung-De Liu, ‘Shi-Rong Qiu and Taida Yiyuan [Shi-Rong Qiu and NTU Hospital], 1964–1972’, *Taiwan Historica*, 59, 4 (2008), 461–2; An-Chiun Chen, *Chen Anjun Huiyilu* [*Memoir of An-Chiun Chen*], Dictated by An-Chiun Chen and Recorded by Xiu-Jen Lin, (Taipei: Wangchunfeng, 2005). For Fu-Min Chen, see Fu-Min Chen, *Laodie de Menzhen Gushi [Stories from Old Daddy’s Clinics]* (Taipei: Peigen, 1999).

23 The most notable practitioner was Dr Chien-Tien Hsu, who established his authority by pioneering a particular method of radical hysterectomy based on the Okabayashi technique. Fu, *op. cit.* (note 13), chapter 5.

24 Fu, *op. cit.* (note 15), 7–8.

25 For example, Dr Hui-Xiong Wu claimed to have delivered 6 000 babies, performed 2 000 surgeries, and conducted 10 000 Pap smears in the course of his career. Hui-Wen Zeng, ‘Dr Hui-Xiong Wu Delivered 6000 Babies and 10,000 Pap Smears’, *Common Health*, 199 (2015), https://www.commonhealth.com.tw/article/article.action?nid=70252, accessed 1 August 2018. There are numerous other examples, including Chien-Tien Hsu (Qian-Tian Xu), who was known for having performed over 5 000 hysterectomies due to cervical cancer. Xiaozhen Qiu , ‘Jingshi Renshi Zhunzhun Jaiohui: Zhuisi Xu qiantian Jiaoshou Baisui Mingdan [In Memory of Professor Chien-Tien Hsu on his 100 Years’ Birthday]’, Taiwan Yijie, 55, 11 (2012), 607–8.

26 Wen-Long Chen, ‘Taiwan Diqu Zigongjingai Fangzhi de Lichengbei [Landmarks of Cervical Cancer Prevention in Taiwan],’ Taiwan Cancer Society, http://www.ccst.org.tw/know/sense-9.html, accessed 1 August 2018.

27 Yu-Ling Huang, ‘Biopolitical Knowledge in the Making: Population Politics and Fertility Studies in Early Cold War Taiwan’, *East Asian Science, Technology, and Society: An International Journal*, 10, 4 (2016), 377–99.

28 John P. DiMoia, ‘“Let us Have the Proper Number of Children and Raise Them Well!”: Family Planning and Nation Building in South Korea’, *East Asian Science, Technology, and Society: An International Journal*, 2, 3 (2008), 361–80. John P. DiMoia, ‘Providing Reassurance and Affirmation: Masculinity, Militarization, and Refashioning a Male Role in South Korean Family Planning, 1962 to the late 1980s’, in Angela Ki Che and Izumi Nakayama (eds), *Gender, Health, and History in Modern East Asia* (Hong Kong: Hong Kong University Press, 2017), 244–69.

29 For a discussion of the work done by social scientists on reproductive technologies in East Asia, see Chia-Ling Wu (Jia-Ling Wu), et al., ‘Gender and Reproductive Technologies in East Asia: A Partial Bibliography of Works in English’, East Asian Science, Technology, and Society: An International Journal, 2, 3 (2008), 327–34.

30 For an overview of the research on the history of cancer medicine, see David Cantor, ‘Introduction: Cancer Control and Prevention in the Twentieth Century’, *Bulletin of the History of Medicine,* 81, 1 (2007), 1–38.

31 Francesca Bray, ‘Introduction’, in Angela Ki Che Leung and Izumi Nakayama (eds), *op. cit.* (note 28), 1.

32 For a study on the era of ‘uncontrolled and high fertility’, see Chi-Hsien Tuan, ‘Reproductive Histories of Chinese Women in Rural Taiwan’, *Population Studies*, 12 (1958), 40–50. For a history of family planning in Taiwan, see Chao-Nan Chen (Zhao-Nan Chen), De-Hsiung Sun (De-Xiong Sun) and Tung-Ming Lee (Dong-Ming Li), *Taiwan de Renkou Qiji* [*The Population Miracle of Taiwan: A study on the Success of the Family Planning Policy*] (Taipei: Linking Publishing, 2003); Kuo, *op. cit.* (note 14). For birth control efforts before State family planning, see Yu-Nong Zhao, ‘Yige Nuxing Jingyan de Jiating Jihua: Taiwan Jiating Jihua Zaoqi de Fazhan [A Female Experience of Family Planning: The Early Development of Family Planning in Taiwan], 1954–1964’, (MA thesis; Taipei: Graduate Institute of History, National Chengchi [Zhengzhi] University, 2004).

33 Zichiu Xu et al., *Jieyu Jingyan* [Experiences of Limiting Births] (Taipei: Central Daily, 1968). The book is a collection of 50 essays written by women on their experiences of limiting births. Most of the authors were professionals.

34 ‘Jihao Lepu zui shihe Taiwan Funu [What size of the Loop is best for Taiwanese women]’, *Jiating Jihua Tongxun [Family Planning Newsletter]*, 2, 3 (1975), 1.

35 The abortion rate among married women was 9% in 1965; by 1973 it had increased to 20%, and in 1980 to 23%, even though it did not become legal until 1984. Hui-Sheng Lin, ‘Taiwan Diqu Youou Funu dui Duotai de Taidu ji shixing Duota de Qushi [The attitudes toward abortion and the trend of abortion among married women in Taiwan]’, *Taiwan Gonggong Weisheng [Taiwan Public Health]*, 8 (1981), 63–80.

36 According to Freedman and Takeshita, ‘Sterilization tends to be reserved for those financially better off, because of its cost.’ Ronald Freedman and John Y. Takeshita , Family Planning in Taiwan: An Experiment in Social Change (Princeton, NJ: Princeton University Press, 1969), 93.

37

Chiu-Mei Tseng (Qiu-Mei Zeng) and Mei-Chi Yu (interviewer and editor), Du Congming yu Wo: Du Shuchun Nushi Fangtanlu [*Tu Tsung-Ming and Me: An Interview with Ms Tu Shu-Chun, dictated by her]* (Taipei: Academia Historica, 2005), 69.

38

Chien-Ming Yu (Jian-Ming You), ‘Riju Shiqi Taiwan de Chanpo [Midwives During the Japanese Occupation]’, Jindai Zhongguo Funu Shi Yanjiu [*Research on Women in Modern Chinese History*], 1 (1993), 49–89.

39

Jin-Xiu Song (ed.), Rizhi Taizhong Funu de Shenghuo [Lives of Women in Taichung during Colonial Period, Oral History Series of Taichung County] (Fengyuan: Cultural Center of Taichung County, 2000), 78.

40 Diary of Xin-Rong Wu, 20 June 1950, Taiwan Diary Database, Institute of Taiwan History, Academia Sinica, http://taco.ith.sinica.edu.tw/tdk/%E9%A6%96%E9%A0%81, accessed 9 February 2015.

41 According to the prominent ob/gyn doctor, Chen Fu-Min, the 1950s was ‘a time of poverty and there was a lack of resources’, including anaesthesia, blood-transfusion, drugs, instruments and manpower, and there was not enough knowledge and training. Therefore, even the ‘simplest’ procedures like C-section and hysterectomy were seen as daunting. Fu-Min Chen, ‘Taiwan Fuchanke Bainian Shi Xilie: Guofang Yixueyuan Pian [A Hundred Years of Obstetrics and Gynecology in Taiwan: The Case of National Defense Medical College]’, *Newsletter of the Obstetrics and Gynecological Association of Taiwan*, 12 (1997), 15–6. The decline of mortality rate for C-section allegedly made it obstetricians’ ‘favorite weapon’. Xi-Yao Chen, ‘Chankeyishi zuixihuan yong de Wuqi [Obstetricians’ favorite Weapon]’, *Dangdai Yixue [Contemporary Medicine]*, 3, 10 (1976), 900–901.

42 Letter from Yaoze Lin answered by Taiwan Provincial Taipei Medical Vocational High School, *Lianhe Bao*, 18 December 1951.

43

George P. Cernada (ed.), Taiwan Family Planning Reader: How a Program Works (Taichung: Chinese Center for International Training in Family Planning, 1970).

44 Wei Sheng Shu [Department of Health], *Weiyu Choumou [Saving for a rainy day]* (Taipei: Weisheng Shu, 1973). To ensure that every junior high graduate had a copy of the booklet, the number of copies matched the number of graduating junior high students each year, approximately 400 000. The first edition was published in 1971, the year the first group of students completed the 9-year national compulsory education that had started in 1968.

45 Taiwan Sheng Jiating Jihua Yanjiusuo [Research Institute for Family Planning for the Province of Taiwan], *Xinhun Jiating Jihua Shouce [Family Planning Manual for the Newly Married]* (Taichung: Taiwan Sheng Jiating Jihua Yanjiusuo*,* 1975).

46 Chen, Sun, and Lee, *op. cit.* (note 32), 159. See also Freedman and Takeshita, *op. cit.* (note 36), 37–67.

47
*Lianhe Bao*, 20 May 1964.

48 The total number was 2 712 277. In addition to IUDs (1 743 532, 64%), others included the pill (518 093, 19.1%), condoms (370 294, 13.65%), vasectomy (6 634, 0.24%) and tubal ligation (73 724, 2.71%). Chen, Sun and Lee *op. cit.* (note 32), 206.

49 Kuo, *op. cit.* (note 14)

50

Zhenzhen Furen [Madame Zhenzhen], ‘Qing anxin fuyong Koufu Biyunyao [Please Take the Pill with Ease]’, Fengnian [Harvest], 23, 12(1973), 50.

51 Feng-Luan Chen, ‘Koufu Biyunyao Shiyonglu di de yuanyin hezai [Why the Rate for Taking the Pill is Low]’, *Fengnian*, 16 July 1971, 37.

52 John P. DiMoia, ‘Providing Reassurance and Affirmation’, *op. cit.* (note 28).

53 The combined number of vasectomies for the period of 1972–1976 was 6 634, as opposed to 73 724 for tubal ligation. In 1983, the reported number of women who were sterilised in Taiwan was 50 100, as opposed to only 3 000 men. John A. Ross, Douglas H. Huber and Sawon Hong, ‘Worldwide Trends in Voluntary Sterilization’, *International Family Planning Perspectives*, 12 (1986), 35.

54 Chen, Sun, and Lee, *op. cit.* (note 32), 206.

55

Bi-Yu Huang , ‘Chi Gongkai de Mimi [A Late Open Secret]’, Fengnian, 22, 11(1972), 54.

56 Yu-Ling Huang, ‘Modernizing Fertility: International Network of Social Knowledge, Contraceptive Technologies, and Family Planning Program between Taiwan and the United States,1950s–1960s’ (PhD dissertation, Department of Sociology, Graduate School of Binghamton University, State University of New York, 2014).

57 A survey done in the period between 1954 and 1958 on 6 517 people indicated that only 27.79% were not practicing any form of birth control, and the reasons were breastfeeding, old age, in postpartum period, and husband’s unwillingness to cooperate. Zhao, *op. cit.* (note 32), 68.

58 ‘Woguo Funu shiyong Lepu Geguo gongren Qingkuang zuijia Wei Bing-Yan Ruishi guilahou xuanbu [Upon returning from Switzerland, Dr Bingyan Wei announced that all nations agreed that Taiwanese women adapted to the Loop best]’, *Lianhe Bao*, 3 January 1968. However, Dr Wen-Long Chen who participated in the family planning pointed out the actual number of perforations of uterus was higher than what Dr Wei claimed, which was one in ten thousand. Wen-Long Chen, ‘Yili Jiaji Zhongzi de Huigu [Reminiscence of a Seed of Family Planning]’, in *Xinhuo Xiangchuan: Taiwan Fuyou Weisheng Xiehui Sishinian Jinian Tekan [Forty Years Anniversary of Planned Parenthood Association of Taiwan]*, 1964–2004 (Taipei: Planned Parenthood Association of Taiwan, 2004), 55.

59 A study indicated that for the three different sizes of the Loop, the rejection rate after 24 months was 50.6%, 49.9%, and 56.6%. ‘Jihao Lepu zui Shihe Taiwan Funu [Which size of the Loop suits Taiwanese Women best]’ *Jiating Jihua Tongxun [Newsletter of Family Planning]*, 2, 3 (1975), 1.

60 For a history of the Lippes Loops in Taiwan, see Nian-Xuan Lu, ‘Lepu Yanjiu: Taiwan Jiating Jihua xia Lepu xiangguan Lunshu yu Nuxing Shenti Jingyan [A Study of Lippes Loop: Discourses on Lippes Loop in Taiwan’s Family Planning Program and Female Body Experiences], 1960s–1980s’, (MA thesis, Kaohsiung: Graduate Institute of Gender Studies, Kaohsiung Medical University, 2010).

61 The number for 1977 was 109 722/755 465, and 656 680/1 628 254 for 1990. Chen, Sun, and Lee, *op. cit.* (note 32), 248.

62

Dong-Ming Li , ‘Yongjiuxing Biyun: Jiezafa zheng Jiji Tuiguang [Permanent Contraception: Sterilisation is being Promoted Actively]’, Fengnian, 25, 22 (1975), 16.

63

Ning Chang , ‘He Shi-Zhu Nushi Fangwen Jilu [Oral History Interview with Ms Shih-Chu Ho]’, in Chien-Ming Yu (ed.), Taizhong Rongmin Zongyiyuan Sanshizai Koushu Lishi Huigu Xiapian [Thirty years at Taichung Veterans General Hospital: Oral Histories, Volume III] (Taipei: Institute of Modern History, Academia Sinica, 2014), 31

64

Zi-Yao Li , ‘Shuruanguan Jieza [tubal ligation]’, Jiankang Shijie [Health World], 23 (1977), 7–9.

65 Chien-Dai Chiang (Qian-Dai Jiang), Chang-E Xu and Pei-Hua Wu, ‘Taipei Shi Funu Jieshou Nuxing Jieza Shoushu zhi Yanjiu [A Study on Women Who Accepted Female Sterilisation in Taipei]’, Funu zai Guojia Fazhan Guocheng zhong di Juese [Women’s Role in the Process of National Development], Taipei, 1985.

66 Yan Qiu, ‘Wode Jingyan [My Experience]’, *Minsheng Bao*, 1 November 1983.

67 Chiang, Xu, and Wu, *op. cit.* (note 65), 4.

68 Tubal ligation via the abdomen was promoted as opposed to tubal ligation via the vagina, because the latter would not allow the surgeon to do an appendectomy during the operation. Xingzheng Yuan Jinghehui [Economic Council of the Administrative Yuan] and Joint Commission on Rural Reconstruction, *Jiating Jihua* [Family Planning] (Council of Family Planning Promotion, Taiwan Sheng Zhengfu Weishengchu, c.a. 1964); Institute of Family Planning, Taiwan Province, *Xinhun Jiating Jihua Shouce [Family Planning Manual for the Newly Married]*, (Nantou: Institute of Family Planning, 1975), 14.

69 Interview with Mrs Xu.

70 This article seems to be a translation from English sources, as there are several terms in English provided in parentheses in the text. Unfortunately, I was not able to identify the original. It nevertheless indicates that it was a shared attitude in the ob/gyn community.

71

Hai-Tao Zhao , ‘Tianshi zhi Ge’ [Angel’s Songs], Wenyi, 94 (1977), 44–86, 63.

72 For example, looking at the medical records of Xu Waike [Xu’s Surgical Hospital], a local hospital established in Kaohsiung in 1956 that specialised in surgery, for the period between August 1958 and July 1959, the total number of surgeries were 1 044, the number-one being appendectomy (248 cases). Kaohsiung City Medical Association (ed.), *Kaohsiung Yiliao Shi [Medical History of Kaohsiung]* (Kaohsiung: Kaohsiung City Medical Association, 1998), 142.

73 Dong-Hui Gao, ‘Chusheng Zhidu Kaipian Hengchun Bandao’ [A Novice Who Cut All Over Hengchun Peninsula], *Hengchun Ren*, 14 (2010), 43.

74 Xin-Xing Zhang, MD, ‘Zigong qiechu hou’ [After Hysterectomy], 84 in An-Chiun Chen, MD, (ed.), *Xiandai Funu Baojian [Keeping Health for Modern Women]* (Taipei: Funu Jiating Chuban She, 1986).

75 The ten conditions were uterine cancer, myoma, choriocarcinoma, endometriosis, uterine rupture, uterine atony, placenta increta, placental abruption, uterine prolapse and sterilization. Chen, *op. cit.* (note 74), 14–15.

76 For example, Zhong-Xiu Ou’s essay, ‘Talking about Hysterectomy,’ originally published in *Yinger yu Muqin Zazhi [Baby and Mother Magazine]* in 1977, in which he writes that ‘the uterus is just a place for nurturing the fetus. When you are old or do not plan to have more children, the uterus no longer serves a function.’ Chen, *op. cit.* (note 74), 14.

77

Xiang-Da Wu (trans.), Ni Xiang Zhidao de Aizheng Zhishi [The Knowledge You Want to know about Cancer] (Taipei: Zonghe Yuekan She, 1973), 74 . The book is a translation of Joseph C. Maroon, *What You Can Do About Cancer* (New York, NY: Doubleday, 1969).

78 Boston Women’s Health Collective, *Our Bodies, Ourselves* (New York: Simon and Schuster, 1976[1971]), 148.

79 Interview with Ms Xu, Kaohsiung, 13 May 2010. By the late 1970s, in addition to being a symbol of medical progress, the practice of hysterectomy had assumed one more characteristic – it was good business. All the women family members of Dr X, an ob/gyn of Kaohsiung city, underwent hysterectomy. Dr X’s practice of hysterectomy flourished during the 1970s and 1980s in Kaohsiung city, and he was known to recommend hysterectomy to most of the women who went to his clinic. Interview with ob/gyn Dr L, Kaohsiung, 11 July 2012; Interview with ob/gyn Dr K, Kaohsiung, 21 April 2017.

80 Physicians had access to new technologies and the novel, attractive ways of living brought about by such technologies. Ming-Cheng M. Lo , Doctors within Borders: Profession, Ethnicity, and Modernity in Colonial Taiwan (Berkeley, CA: University of California Press, 2002).

81 Before the 1970s, little distinction was made between uterine and cervical cancer, and considering the fact that uterine cancer was generally much less prevalent, uterine cancer often referred to cervical cancer.

82 Shu Yeh (Shu Ye) and E. V. Cowdry, ‘Incidence of malignant tumors in Chinese, especially in Formosa’, *Cancer,* 7 (1954), 425–436. The article also refers to early studies in the 1920s and 1930s that made the same observation – in women cervical cancer was the most prevalent tumour.

83 National data on cervical cancer from the early post-Second World War period do not exist. Department of Health, Taiwan Provincial Government, *Vital Statistical Abstract of Taiwan Province*, 1970. Pesus Bise Chou , ‘Zhongmei Liangguo de Aizheng Tongji [Cancer Statistics for the US and Taiwan]’, Taiwan Yijie [Taiwan Medical Journal], 20, 2 (1977), 28–33.

84 For example, *Shuizhuju Zhuren Riji [Diaries of Lijun Zhang]*, 15 January 1929, 22 May 1933, 3 October, 8 October 1933, 19 December 1933; 10 May 1934, 2 July 1934, 17 July 1934, 1 August 1934; 20 September 1935, in which three women in Zhang’s social network suffered from zigong bing (disease of the uterus) and received treatments such as surgeries and radiation.

85

Pesus Bise Chou (Bi-Se Zhou), ‘Zhongmei Liangguo de Aizheng Tongji [Cancer Statistics of the Republic of China and the US]’, Taiwan Yijie, 18, 2 (1977), 31.

86

Pesus Bise Chou (Bi-se Zhou), ‘Zhongmei Liangguo de Aizheng Tongji [Cancer Statistics of the Republic of China and the US]’, Taiwan Yijie, 19, 7 (1976), 26–31.

87

Chiu Yen (trans.), ‘Public Enemy for all Women—Uterine Cancers’, Weisheng Zazhi, 7 (1952), 15–17.

88

Chi Zhuang , ‘Zigongai [Uterine Cancer]’, in Fuer Bing Fanghu Shouce [Prevention and Care Manuals for Women’s and Children’s Diseases] (Taipei: Hung Yeh Publishing, 1966), 85 . *Jiehun yu Xin Yixue Shidian [Manual for Marriage and Sexual Health]* (Taichung: Jingwen Shuchu, 1964), 153.

89 Dr Esther Shih-Chu Ho (Shi-Zhu He), ‘Funu de Touhao Diren: Zigongjingai [The Number One enemy of Taiwanese women: Cervical Cancer’, *Cancer Bulletin of S.Y. Dao Memorial Fund*, 3 (1997), 30–33.

90

Julia Tsuei and Tao-Sun Wang , ‘Dingqi Lixing Yindao Mopian Xibaoxue Yanjiu dui Faxian Zaoqi Zigongjingai zhi Chengxiao [Detection of early cervical cancer by routine pap smear screening]’, The Journal of Obstetrics and Gynecology of The Republic of China (Taiwanese Journal of Obstetrics & Gynecology), 12, 4 (1973), 164–171.

91

Kazumasa Masubuchi , Yoshio Tenjin , Jun-Ichi Fujii , ‘The detection of Cervical Cancer at Cancer Hospital in Tokyo from 1952 to 1963’, Journal of Clinical Cytology and Cytopathology, 11, 1 (1967), 32–4.5233137

92 Fu, *op. cit.*, (note 13), chapter 4.

93 Dr Yu-He Cheng recalled his experience in 1948 when 250 women were admitted to the Central Hospital in Nanking in Mainland China, and among them 30 were cervical cancer cases. The limited quantity of radium available kept them from admitting more patients. Yu-He Cheng, ‘Tan Ai Sebian’ (Turning Pale when Speaking of Cancer) in Yung-Chun Liu, MD (ed.) *Jibing Yu Yiliao [Diseases and Medicine]* (Taipei:Zhongwai Chuban She, 1949), 98–100.

94

Manqing Xiao , Jifeng Jincao: Xiangwo Zheyang de Muqin [The Grass Standing the Trial of Storms: A Mother Like Me] (Taipei: Jianxing Wenhua, 2014), 343.

95 Hsiao-Ling Chen (Xiao-Ling Chen), ‘Qiongkun de Mama yu Xinshidai Shaonu: Zigong Jingai Fangzhi de Meiti Lunshu [From Poor Mothers to Modern Young Girls: The Changing Image of High Risk Groups for Cervical Cancer in Taiwan], 1960s–2000s’, (MA thesis, Kaohsiung: Graduate Institute of Gender Studies, Kaohsiung Medical University, 2008).

96 ‘Jiubing nan Zhiyu Duzi Fu Zisha Linnei Laofu chu ci Xiace [Old woman poisoned her son and then committed suicide as a result of suffering from incurable disease]’, *Lianhe Bao*, 28 April 1961.

97 Hua Yen, ‘Wo Shang Shoushu Tai’ (I got on the Operating table), in *Hua Yen Duanwenji [Collection of Hua Yen’s Short Essays]* (Taipei: Yao-sheng, 1991), 244. The essay was originally published in *Lianhe Bao*, 7 July 1965. Hua Yen is a pen name of Ting-Yun Yen (b. 1926).

98 Chun-Can, ‘Zigongjing ai duozou le wode qizi’, (Cervical cancer took away my wife), in Shi-Zheng Li (ed.) *Jiuyi Jingyan Tan [Experiences of Seeking Medical Help]*, 159–174. Shao-Zhen Jiang, *Jiang Guei-qin de zhenshi Gushi: Wo dui Ainu de Huiyi [The True Story of Guei-Qin Jiang: Memories of My Beloved Daughter]* (Taichung: Mingguang Chubanshe, 1973).

99

Tien-Yu Lin , Xinglin Shengya Yun he Yue [Life of being a Surgeon] (Taipei: Commercial Press, 1990), 91–103.

100 ‘Wang Taitai Xinzhong de Kongju [The Fear in Mrs Wang’s Heart]’, *Jiankang Shijie*, 3 (1976), 52–3.

101

Zhong-Quan Zhang , ‘Funu zhi Dadi: Zigongjingai [Women’s Big Enemy—Cervical Cancer]’, Baby and Mother, 23, 7 (1978), 15.

102

Zhong-Quan Zhang , ‘Taiwan Zigongjingai de Fangzhi [The Prevention of Cervical Cancer in Taiwan]’, Kexue Yuekan [Science Monthly] 40 (1973), 48–52.

103 ‘Taida Yiyuan Fuke Zengshe Zigongai Tebie Menzhenbu [Establishing a Special Clinic for Uterine Cancer at the Gynecological Department, NTU Hospital]’, *Lianhe Bao*, 6 December 1953. According to the report, quoting Dr Shi-Rong Chiu [Shi-Rong Qiu], in the period between 1938 and 1950, there were 741 cases of women’s cancer, and 17% were in the first stage.

104 Löwy, *op. cit.* (note 16).

105 Some success stories of early detection began to appear. Xing-Jie Rao, a patient who received hysterectomy in 1976, wrote that ‘Uterine Cancer No Longer Threatens Me’, and, as she had caught it early enough, she hoped for a 100% cure. Xing-Jie Rao (b. 1940), ‘Zigong Ai buzai Weixie Wo’ [Uterine Cancer No Longer Threatens Me], in *Jiankang Xuanji [Essays on Health]*, vol. 1 (Taipei: Zhongyang Ribao She, 1979).

106 Ob/gyns such as Yi-Hung Zhan also shared the same view. Yi-Hung Zhan, ‘Yige Fuchanke Yishi de Youlu [An Ob/gyn’s worries]’, *Funu Xinzhi Zazhi [Awakening Magazine]*, 5 (1982), 22–4.

107

Alexandra Howson , ‘Surveillance, Knowledge, and Risk: The Embodied Experience of Cervical Screening’, Health, 2, 2 (1998), 195–215.

108 Leslie J. Reagan, ‘Engendering the Dread Disease’, *American Journal of Public Health*, 87 (1997), 1779–1787. For a comparative study on the dread of cancer, see James T. Patterson, ‘Cancer, Cancerphobia, and Culture: Reflections on Attitudes in the United States and Great Britain’, *Twentieth Century British History*, 2, 2 (1991), 137–49.

109

Pesus Bise Chou , ‘Shengsi zhijian [Between Life and Death]’, Taiwan Yijie, 20, 1 (1977), 1.

110

Pesus Bise Chou , ‘Fangai Xiehui Fuwu Baogao (7) [Report of the Itinerary Cancer Prevention Service—7]’, Taiwan Yijie, 20, 1 (1977), 45 .

111 Charles Rosenberg, ‘Managed Fear’, *The Lancet*, 373 (2009), 802–3; Anne M. Kavanagh and Dorothy H. Broom, ‘Embodied Risk: My Body Myself?’, *Social Science and Medicine*, 46, 3 (1998), 437–44.

112 Pesus Bise Chou, ‘Fangai Fujian Zuotanhui [Cancer Prevention and Rehabilitation Forum]’, *Taiwan Yijie*, 20, 6 (1977), 35.

113 The rate of suspicious results during the first mass screening in the period between 1974 and 1979 was 1.30% (among 77 599 women tested), and the estimated cancer rate was 0.60%. Pesus Bise Chou and Vivien Chen, ‘Mass screening for cervical cancer in Taiwan from 1974 to 1984’, *Cancer*, 64 (1989), 962–8.

114 Chou, *op. cit.* (note 111).

115 Interview with Ms Huang, Kaohsiung, 22 July 2008.

116 Chou and Chen, *op. cit.* (note 113); Kangchu Zhu, ‘Zhongguo Funu Zigongjingai zhi Yanjiu [A study on Chinese women’s cervical cancer]’, Research Report NSC 65-0421-B016-35, 1976.

117 Pesus Bise Chou, ‘Fangai Xiehui Fuwu Baogao [Report of the Itinerary Cancer Prevention Service]’, *Taiwan Yijie*, 10 (1977), 32–4.

118 Tao-Sun Wang, ‘Yufang Zigongai Yao Dingqijiancha [To Prevent Uterine Cancer, Regular Exams Are Necessary]’, *Fengnian*, 27, 9 (1977), 49–50.

119 Fu, *op. cit.* (note 13), 112–113.

120 Rui-Mei Yang, ‘Fangshi Huaxu [Visiting Titbits]’, *Fengnian*, 17, 5 (1968), 53.

121

Jing-Jing Furen [Madam Jingjing], ‘Jiating Jihua Xinxiang [Family Planning Mailbox]’, Fengnian, 19, 24 (1969), 48.

122 Wang, *op. cit.*, (note 17).

123 For example, a news report quoted uterine cancer authority, doctor Junxiong Wang’s warning to women. ‘Zigong Chuxie Baidai duo shi Zigongai Yuzhao [Uterine bleeding and Whites Are Signs of Uterine Cancer]’, *Lianhe Bao*, 17 April 1959.

124 A free pamphlet for women distributed by a Chinese herbal company quotes a National Taiwan University Hospital’s report on uterine cancer as its source of authority and concludes: ‘The early symptoms of uterine cancer are irregular menstruation and *chibaidai* (red and whites).’ Da-Hai Wu, *Nuxing de Jianmei Yu Shengli Weisheng [Women’s Healthy Beauty and Hygienic Physiology]* (Chiayi: Jinan Zhiyaochang,1959), 15–6.

125 Chen, *op. cit.* (note 93).

126 The second doctor was perplexed that a hysterectomy was recommended without any diagnostic procedures. Sumama mentioned that the uterus of most of her old women colleagues at the elementary school had been removed. Interview with Ms Xiao, Kaohsiung, 25 April 2012. Interview with Sumama, March 2007; August 2010. Ms Xiao also heard very similar stories. Interview with Ms Xiao, Kaohsiung, 25 April 2012.

127

Sheng-Ping Zhang , Yao-Xun Lai and Xiang-Da Wu , ‘Quan zigong qiechu shoushu – 1499 bingli fenxi [Total Hysterectomy—An analysis on 1499 cases]’, Linchuang Yixue [Clinical Medicine], 5 (1980), 122–8.

128 Xin-Ru, ‘Weihe shixing zigong qiechu Shu? [Why Perform the Procedure of Hysterectomy],’ *Dazhong Yixue*, 17, (1967), 332.

129 Women diagnosed with a myoma might undergo the agonizing experience of not knowing what exactly was growing inside the uterus. See, for example, Hui-Han Wang, ‘I have uterine myoma’, in Shizheng Li (ed.), *Jiuyi Jingyan Tan [Medical Experiences]*, (Taipei: Minsheng Daily Publishing, 1987), 153–5. The essays were originally published in *Minsheng Ribao* in 1983.

130 Another example is Mrs Wang. After telling her that she had a myoma, the doctor forewent any diagnostic examinations and hastily recommended hysterectomy. He scheduled the surgery for the following morning. Interview with Mrs Wang, 12 August 2010.

131 Tsai and Wang, *op. cit.* (note 15).

132 Zhang, *op. cit.* (note 82).

133 Dr Xi Cun, *Xin Yixue Baike Wenda [Encyclopedic Q and A for Sexual Medicine]* (Taipei: Xiansheng Chuban She, 1973), 263–4.

134 Newspapers’ health advice columns told of the risk of not removing the ovaries when doing a hysterectomy, implying that by accepting the surgery (hysterectomy plus ovariectomy) a woman would be free from three types of cancer – cervical, uterine, and ovarian. *Lianhe Bao*, 21 April 1976.

135 Yi-Hung Zhan, ‘Chufei biyao qing buyao qiechu zigong [Unless It’s Necessary, Please Do Not Cut Off Your Uterus]’, in *Fuke: Cong Qingchunqi dao Gengnianqi Yiliao Zhinan [Gynecology: Medical Guidance from Adolescent to Menopause]* (Taipei: Shibao, 1994), 318–9.

136 Dr Lan’s translation of information from the American Cancer Society, *Lianhe Bao*, 23 June 1970.

137 The article did not provide the original English for the name Lu Ka Si. Li Ming (trans.), ‘Renshi Luanchaoai [Understanding Ovarian Cancer]’, *Lianhe Bao, 21* April 1976.

138 For example, Lin (surgery date 1997), Mrs Wang (1991) and Ms Wu (1990).

139 Public Employees Insurance Law, Article 3 and Labor Insurance Act, Article 63.

140 According to Article 3 of GEI, a woman under 45 who loses fertility due to hysterectomy, ovariectomy, or cancer treatment, is qualified to receive cash benefit of 6–8 months of her salary. Article 3 was revised in 2000 to prevent unnecessary hysterectomy by adding a proviso that inappropriate and prophylactic hysterectomies are not qualified for the benefit.

141 Central Trust of China, *Statistical Data for Government Employees’ Insurance*, Republic of China (1991). However, even though these early statistics included the total number of the insured persons, there was no gender ratio available until 1999.

142 The number of insured persons was 172 469 in 1958, 189 271 (1959), 203–909 (1960), 213 419 (1961), and by 1990 it was 1 078, 449. The gender ratio is unknown. The hysterectomy rate, if calculated by 100 000, was 1.47 in 1960, 3.27 in 1961, and by 1962 it had become 28.84.

143 Labor Insurance Act, Article 63 in 1958, Article 64 in 1968, Article 53 in 1979, in section 5 Permanent Disability Benefits.

144 One author concludes, ‘Never remove the Uterus casually, as any surgery carries a certain degree of risk.’ Another similar essay, a medical column in *Dangdai Yixue Zazhi [Contemporary Medicine Magazine]*, concludes, ‘Some thought older women after giving birth may accept hysterectomy. This is excessive and unnecessary’. Zuo Rong, ‘Renshi Zigong Jiliu [Understanding Uterine Myoma]’, *Lianhe Bao*, 15 August 1973; *Lianhe Bao*, 11 June 1975. Jia Jin (trans.), ‘Yishi buyinggai qishi Nuxing Bingren [Physicians Should not Discriminate against Female Patients]’, Zhonghua Funu Yuekan [China’s Women Monthly], 25, 1, (1974), 16.

145

Xiang-Da Wu , ‘Weishenme Qiechu Zigong [Why hysterectomy]?’ Funu Zazhi, 4 (1976), 81–83.

146 Zhang, Lai, and Wu, *op. cit.* (note 125), 128.

147 ‘Yisheng huluan huibairen binghuan mangran aizaige mangchang zigong qieliao zaishuo mingli shuangsho hele buwei [Doctors wave unruly knife at Unsuspecting Patients; Appendix or Uterus, Cut them first. Why not do it for Fame and Money?]’ *Lianhe Bao*, 7 October 1977.

148

Zu-Miao Zhao , Choulou de Yisheng [Ugly Doctors] (Taipei: Siji Publishing, 1978)

149 Bo Yang [Ding-Sheng Guo], ‘Kangzai Weiyuanhui [Resisting Butchery Council]’, in Bo Yang, *Huogai Ta He Luojiang* (Taipei: Yuanliu, 2000), 100–4. The essay was originally published in 1977 in *Zhongguo Shibao [China Times].*

150 Wan-Fen Tsai (Wan-Fen Cai), ‘Women’s Health Status as seen through Women’s Health Service Support Hot Line’, http://newcongress.yam.org.tw/women/99inf020.htm, accessed 21 April 2017.

151

Robert A. Aronowitz , ‘Do Not Delay: Breast Cancer and Time, 1900–1970’, Milbank Quarterly, 79 (2001), 355–386.10.1111/1468-0009.00212PMC275120211565161

152 Warwick Anderson, ‘Afterword: Biomedicine in Chinese East Asia: From Semicolonial to Postcolonial?’, in Angela Ki Che Leung and Charlotte Furth eds., Health and Hygiene in Chinese East Asia: Policies and Publics in the Long Twentieth Century (Durham, NC: Duke University Press, 2011), 273–8.

153 Charlotte Furth and Shu-Yueh Chen, ‘Chinese Medicine and the Anthropology of Menstruation in Contemporary Taiwan’, *Medical Anthropology Quarterly*, 6, 1 (1992), 27–48; Hsiu-Yun Wang , ‘Postcolonial Knowledge from Empires: The Beginnings of Menstrual Education in Taiwan, 1950s–1980s’, East Asian Science, Technology and Society: An International Journal, 11, 4 (2017):1–22.

154 Fu, *op. cit.* (note 13).

## Appendix

Interviewees (all the names are pseudonyms).

**Table 1:**

Name	Born	Location	Birth control	Hysterectomy	Reasons	Removed	Interview date
Mrs Lai	1952	Pingtung	Unknown	1997	Myoma, heavy bleeding	uterus	10 April 2009
Mrs Qiu	1941	Pingtung	None	1987	Pain, myoma	uterus	10 April 2009
Ms Miao Young	1952	Miaoli	Contraceptive	1990s	Myoma, bleeding	uterus	29 May 2010
Mrs Miao	1942	Miaoli	Lippes Loop	1972	Leucorrhoea, fear of cancer	uterus	29 May 2010
Ms Xu	1951	Kaohsiung	Lippes Loops	1997	Menstruation	uterus	13 May 2010
Ms Wu	1961	Pingtung	Tubal ligation	1991	Menstrual pain, myoma, fallopian tube oedema	uterus	11 Aug 2010
Mrs Lin	1950	Pingtung	Tubal ligation	1997	Myoma, oedema, fear of cancer	uterus, ovaries	11 Aug 2010
Mrs Wang	1954	Taichung	Tubal ligation	1991	Myoma	uterus, ovaries	12 Aug 2010 phone
Mrs Moon	1960	Taichung	Tubal ligation	1997	6–7 cm myoma	uterus	12 Aug 2010 phone
Lin Spring	1954	Kaohsiung	None	2003	6–7 cm myoma	uterus	13 Aug 2010
Zheng Snow	1958	Pingtung	None	1990	Myoma	uterus	14 Aug 2010 phone
Ms Huang	1946	Pingtung	None	1981	Myoma, heavy bleeding	uterus, ovaries, appendix	4 July 2012
Mrs Cai	1942	Taichung	Lippes Loops	1990	Suspicion of cervical cancer	uterus	5 Sep 2012
Sumama	1932	Pingtung	None	1981	Declined the surgery	NA	12 Aug 2010

Medical practitioners: Ms C (midwife) Hualien, interviewed on 2 August 2011; Dr L (ob/gyn since 1980) Kaohsiung, interviewed on 11 July 2012; Dr J (family practitioner since 1985) Kaohsiung, interviewed on 24 May 2012; Dr K (ob/gyn since 1973) Kaohsiung, interviewed on 21 April 2017.

